# Dynamic spin reordering in a hybrid layered ferrimagnet with intercalated biferrocenium radicals†

**DOI:** 10.1039/d4sc04722b

**Published:** 2024-10-30

**Authors:** Qingxin Liu, Wataru Kosaka, Hitoshi Miyasaka

**Affiliations:** a Institute for Materials Research, Tohoku University 2-1-1 Katahira Aoba-ku Sendai 980-8577 Japan hitoshi.miyasaka.e7@tohoku.ac.jp; b Department of Chemistry, Graduate School of Science, Tohoku University 6-3 Arama-ki-Aza-Aoba Aoba-ku Sendai 980-8578 Japan

## Abstract

Molecule-based hybrid layered magnets provide an ideal platform for investigating the long-range spin-ordering process in low-dimensional magnetic systems. Within this context, a promising area of research is spin-sandwiched hybrid layered magnets. These materials offer the potential to explore how the spin, which is sandwiched between magnetic layers, is influenced by the internal magnetic fields generated by the magnetic layers. Herein, we report a layered ferrimagnet with intercalated biferrocenium ([bifc]^+^) radicals, [bifc][{Ru_2_(2,3,5,6-F_4_ArCO_2_)_4_}_2_(TCNQF_2_)] (1, TCNQF_2_ = 2,5-difluorotetracyano-*p*-quinodimethane). The [{Ru_2_(2,3,5,6-F_4_ArCO_2_)_4_}_2_(TCNQF_2_)]^−^ moiety acts as a ferrimagnetic layer with *S*_T_ = 3/2, composed of a paddlewheel [Ru_2_^II,II^(2,3,5,6-F_4_ArCO_2_)_4_] (2,3,5,6-F_4_ArCO_2_^−^ = 2,3,5,6-tetrafluorobenzoate) with *S* = 1 and 2,5-difluoro-7,7,8,8-tetracyanoquinodimethanate (TCNQF_2_˙^−^) units with *S* = 1/2 in a 2 : 1 ratio. The isostructural paramagnetic compound [bifc][{Rh_2_(2,3,5,6-F_4_ArCO_2_)_4_}_2_(TCNQF_2_)] (2) consisting of diamagnetic [Rh_2_^II,II^(2,3,5,6-F_4_ArCO_2_)_4_] components was also synthesized. An investigation of the properties of 2 revealed minimal magnetic interaction between the [bifc]^+^ and TCNQF_2_˙^−^ components. Compound 1 displayed long-range ferrimagnetic ordering at the Curie temperature of 105 K without any frequency dependence on alternating current (AC) susceptibility, due to the combination of predominant ferrimagnetic ordering within the layer and interlayer ferromagnetic dipole interactions. However, subsequent stepwise magnetic ordering involving a strong AC frequency dependence was observed upon further cooling. These dynamic behaviors are associated with the ordering of two types of anisotropic [bifc]^+^ spins between the ferrimagnetic layers, indicating that [bifc]^+^ spin ordering is sensitive to anisotropic internal magnetic fields generated by the ferrimagnetic layers.

## Abstract 2

A hybrid layered magnet with a free-spin-sandwiched structure was synthesized using biferrocenium cation [bifc]^+^ as the free-spin component, revealing the characteristic magnetic transition nature of the spin-sandwiched magnetic layer.

## Graphical abstract

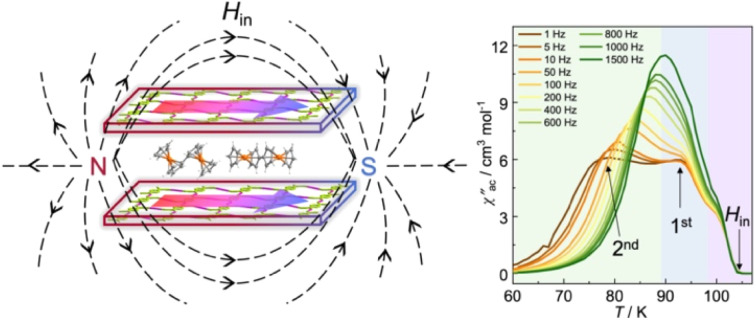

## Introduction

Molecular magnetic materials have the ability to design and create unique combinations of diverse spin structures, enabling the formation of distinct low-dimensional frameworks such as clusters, one-dimensional (1-d) chains, two-dimensional (2-d) layers, and even three-dimensional (3-d) infinite structures. These materials, developed through molecular assembly, engineering,^1,2^ and charge transfer design using a bottom-up approach,^3–8^ exhibit unique physical phenomena such as the spin-Peierls transition,^9^ the superparamagnetism on single-molecule/single-chain magnets,^10–13^ and the Berezinskii–Kosterlitz–Thouless transition.^14^ Thus, this class of low-dimensional magnetic materials is an ideal platform for studying fundamental spin–spin interaction processes.^15^ In particular, magnetic materials with anisotropic 2-d layered structures have garnered significant attention owing to their ability to exhibit long-range magnetic ordering within a single layer.^16–19^ In conventional 2-d layered systems, two types of interactions dominate long-range ordering: one through chemical bonds within the 2-d layer lattice and the other through dipole interactions between the layers. The interaction between these forces determines whether the material exhibits a magnetic phase of either ferromagnet/ferrimagnet or antiferromagnet in the most common 2-d magnets.^20^

**Scheme 1:**
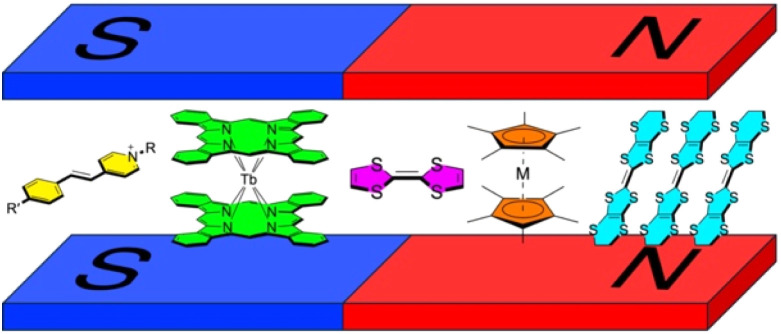
Hybridized layered magnets with intercalated functional molecules including aromatic organic cations, single-molecule magnets, redox active molecules, and conducting molecules. These materials provide a platform for exploring how sandwiched molecules are influenced by the layer-generated *H*_in_.

One of the key features of 2-d magnets is the presence of a strong internal molecular field between the magnetic layers (*i.e.*, internal field, *H*_in_), resulting from long-range ferro- or ferrimagnetic ordering. According to Weiss, this internal phenomenon occurs when a molecular field is assumed inside a ferromagnetic material, often exceeding 1 × 10^3^ Tesla,^21^ a value that is difficult to achieve in a typical laboratory.^22^ Therefore, molecules between magnetic layers are exposed to this strong anisotropic magnetic field, allowing control over their intrinsic properties, such as nonlinear optical properties,^23–27^ single-molecule magnetism,^28^ redox activity,^20,29^ and conductivity^30^ using an *H*_in_ (Scheme 1). In other words, we can investigate the effect of a large *H*_in_ on the physical properties of the encapsulated molecules in a sandwiched magnetic layer without using an external magnetic field. Some related functional molecule-sandwiched layered magnets have actually been reported so far.^30–39^ However, the effects of the internal magnetic field on the free spins of the sandwiched molecules remain largely unexplored.

**Scheme 2:**
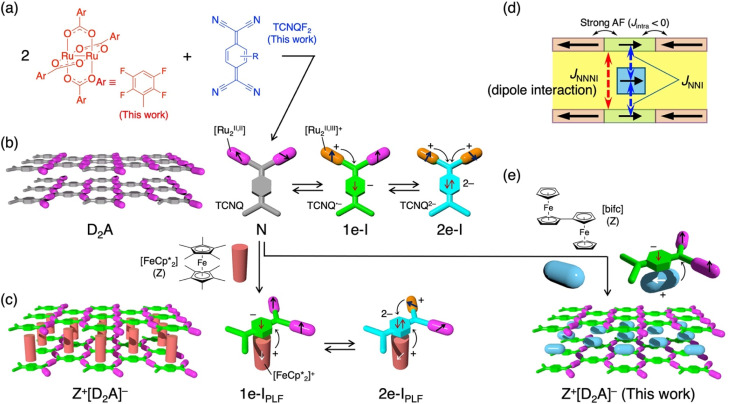
Schematic representations of synthetic routes for D_2_A and Z[D_2_A] and their charge and spin states. (a) Reaction scheme of paddlewheel [Ru_2_^II,II^] complexes and TCNQ in a 2 : 1 stoichiometric ratio, in which [Ru_2_(2,3,5,6-F_4_ArCO_2_)_4_] was used. (b) The charge and spin arrangements of the D_2_A-type. (c) The charge and spin arrangements of the Z[D_2_A]-type, where Z represents 
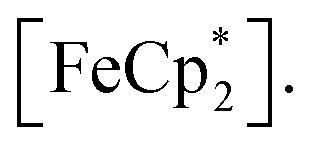
 (d) Spin alignment in a spin-sandwiched hybridized layer magnet, where *J*_NNI_ represents the nearest neighbor interaction, *i.e.*, interaction between [bifc]^+^ and layer, and *J*_NNNI_ represents the next-nearest neighbor interaction, *i.e.*, interlayer inter-unit interaction or dipole interaction. (e) Synthetic scheme for Z[D_2_A]-type with Z = [bifc]^+^ as described in this work.

Our group focused on designing spin-hybridized layered magnets using the family of charge-transfer layered magnets obtained from the reaction of a paddlewheel-type diruthenium(II,II) complex ([Ru_2_^II,II^]) acting as an electron donor (D) and a tetracyano-*p*-quinodimethane derivative (TCNQR_*x*_) acting as an electron acceptor (A) in a 2 : 1 stoichiometric ratio (*i.e.*, D_2_A-type; Scheme 2a).

These systems exhibit three types of steady charge states: D_2_^0^A^0^ (N-state), D_2_^0.5+^A^−^ or D^0^D^+^A^−^ (1e–I state), and D_2_^+^A^2−^ (2e–I state) (Scheme 2b).^3,4^ Among these states, only the 1e–I state allows for long-range ordering of either ferrimagnets or antiferromagnets, depending on the interlayer interaction among prominently ferrimagnetically ordered layers. This type of layered system enables the encapsulation of molecules such as aromatic molecules like pyrene and naphthalene,^40,41^ electron donors like tetrathiafulvalene,^42^ and magnetic molecules such as decamethylmetallocenium cations 
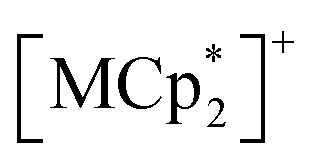
 (M = Co, *S* = 0; Fe, *S* = 1/2; Cr, *S* = 3/2).^20,43,44^ The encapsulation occurs *via* π–π interactions with the six-membered ring of the TCNQR_*x*_ unit and/or the benzoate ligands of the [Ru_2_] units in the layer, thus stabilizing a hybridized layered structure exhibiting quasi-three-dimensional characteristics. These intercalated redox-active molecules strongly influence the charge state and magnetic properties of the 2-d layer.^40^ For example, when 
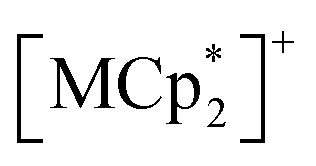
 (abbreviated as Z^+^) is inserted, two distinct charge states Z^+^[D_2_^0^A^−^] and Z^+^[D^0^D^+^A^2−^] are isolated depending on the D unit employed, with only the former exhibiting long-range ordering (Scheme 2c). Specifically, Z^+^[D_2_^0^A^−^] likely forms through a charge-transfer reaction between the N-state of D^0^_2_A^0^ and Z^0^ in a D_2_A-type reaction medium by mixing with another electron donor Z (Scheme 2c). In this hybridized magnetic layer, two types of interactions compete: ferromagnetic interactions between spins of 
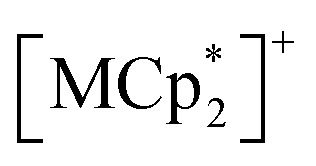
 and TCNQ˙^−^ (the nearest neighbor interaction, *J*_NNI_) and interlayer interactions that can be either ferromagnetic or antiferromagnetic interactions (next-nearest neighbor interaction, *J*_NNNI_), determine the magnetic phase (Scheme 2d).^20,43^ Thus, this type of hybridized magnetic layer serves as a platform that demonstrates the characteristic nature of long-range ordering influenced directly by the insertion of paramagnetic molecules. In fact, it would be nearly impossible to create such a material using conventional magnets, and this is where the advantages of molecular magnets really come into play. Herein, we present two hybrid layered structures that incorporate a biferrocenium cation with an *S* = 1/2 spin state ([bifc]^+^) between the [D_2_A]^−^ layers: [bifc][{M_2_(2,3,5,6-F_4_ArCO_2_)_4_}_2_(TCNQF_2_)] (M = Ru, 1; Rh, 2; 2,3,5,6-F_4_ArCO_2_^−^ = 2,3,5,6-tetrafluorobenzoate, TCNQF_2_ = 2,5-difluorotetracyano-*p*-quinodimethane) (Scheme 2e). These compounds are isostructural and highly stable due to the absence of an interstitial crystallization solvent, even in their as-synthesized form. The neutral, diamagnetic [bifc] acts as an electron donor (Z in Scheme 2e), potentially adopting various electronic states involving charge transfer, such as neutral, valence-site-trapped monocation, valence-detrapped monocation, and dication, similar to charge transfer salts assembled with TCNQR_*x*_.^45–50^ Additionally, [bifc]^+^ with *S* = 1/2 exhibits strong magnetic anisotropy due to spin–orbit coupling.^51–53^ In this context, the [bifc]^+^TCNQF_2_˙^−^ salt (Z^+^A^−^) consistently resides within the Z^+^[D_2_A]^−^ structure, formulated with [M_2_^II,II^] units of D^0^ ([Ru_2_^II,II^], *S* = 1; [Rh_2_^II,II^], *S* = 0). Compound 2 revealed that the [bifc]^+^ spins do not significantly interact with the spins of TCNQF_2_˙^−^. Compound 1 exhibited a magnetic phase transition (*T*_c_) at 105 K due to predominant ferromagnetic coupling between the ferrimagnetic [D_2_A]^−^ layers, *i.e.*, *J*_NNNI_ > 0. However, upon cooling, multiple dynamic spin orderings were observed, attributed to another long-range ordering associated with two types of [bifcs]^+^ spins differently aligned between the [D_2_A]^−^ layers, reflecting the anisotropic spin ordering of the hybridized layer in 1.

The aim of this study was to investigate the influence of *H*_in_ as generated by the magnetic layers on the isolated spin intercalated between them. The study explored the subsequent glassy long-range ordering caused by impact of *H*_in_, which well displays the behavior of isolated spins inside an *H*_in_, being strongly associated with the anisotropy of the intercalated spins.

## Results and discussion

### Synthesis and structural characterization

Taking into consideration the redox potentials of bifc and TCNQR_*x*_, we selected the highly electronegative TCNQF_2_ for these experiments.^48^ Additionally, the [Ru_2_^II,II^(2,3,5,6-F_4_ArCO_2_)_4_] was chosen to stabilize the TCNQF_2_˙^−^ radical form as [bifc]^+^TCNQF_2_˙^−^ and to prevent electron transfer from D to A.^44^ Consequently, the charge state of the products was rationally predicted by balancing the HOMO energy levels of Z and D with the LUMO energy level of A.^54^ Crystalline samples of 1 and 2 were obtained *via* a self-assembly method by adding [M_2_^II,II^(2,3,5,6-F_4_ArCO_2_)_4_(THF)_2_] (M = Ru, Rh) in *p*-xylene solution to a dichloromethane solution containing TCNQF_2_ and bifc. The materials were obtained in yields ranging from approximately 20% to 30%.

**Fig. 1:**
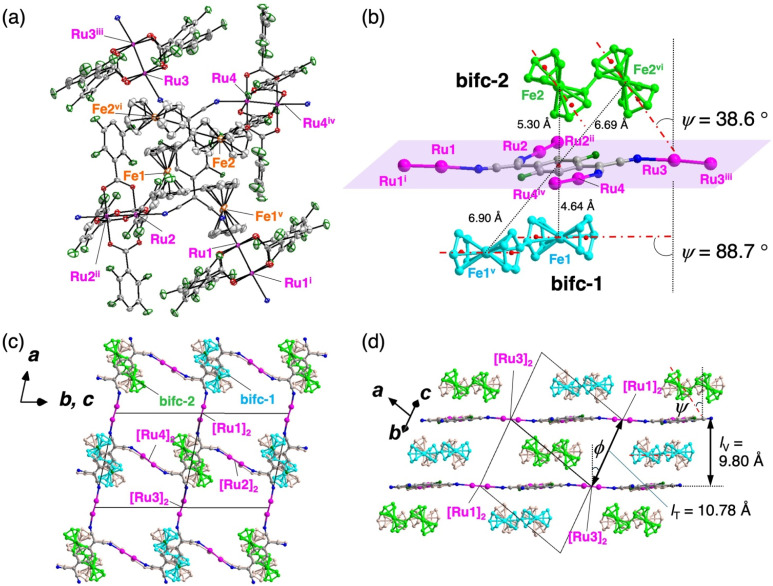
Structure of 1. (a) Thermal ellipsoid plots (with 50% probability) of the asymmetric unit, in which hydrogen atoms are omitted for clarity and O, C, N, F, Fe, and Ru atoms are represented in red, gray, blue, green, orange, and purple, respectively. The symmetry operations are (i) −*x*, −*y*, −*z* + 2, (ii) −*x* + 1, −*y*, −*z* + 1, (iii) −*x* + 2, −*y* + 1, −*z* + 1, (iv) −*x* + 1, −*y* + 1, −*z* + 2, (v) −*x* + 1, −*y*, −*z* + 2, (vi) −*x* + 1, −*y* + 1, −*z* + 1. (b) Closed view around the TCNQ moiety and relationship of two types of [bifc]^+^ units from the layer, where the purple plane represents the (1−11) D_2_A layer and the red dashed-dotted line represents the Fe–Cp_cent_ in [bifc]^+^ (the red dashed-dotted line in (d) is also identical). (c and d) Packing views along the [01−1] (c) and [011] (d) directions, respectively, where the 2,3,5,6-F_4_ArCO_2_^−^ ligands around the Ru centers and all hydrogen atoms are omitted for clarity, and only the forefront subunits are depicted in color. The atomic colors in all panels are the same as those in the panel (a), except for [bifc]^+^; bifc-1 and bifc-2 are colored in cyan and yellow green, respectively.

Single-crystal X-ray diffraction (XRD) analysis conducted at 102 K confirmed that 1 and 2 are isostructural. The purities of the bulk samples were confirmed by examining their powder XRD patterns (Fig. S1†). Compounds 1 and 2 crystallized in the triclinic space group *P*1̄ (no. 2) with an asymmetric unit comprising four halves of [M_2_] subunits, one whole TCNQF_2_, and two halves of bifc (Table S1,† and Fig. 1a and S2a† for 1 and 2, respectively). The inversion centers are located at the midpoint of all [Ru_2_] and bifc subunits, and all the atoms of TCNQF_2_ are crystallographically unique. Two sets of formula units of [bifc][{M_2_(2,3,5,6-F_4_ArCO_2_)_4_}_2_(TCNQF_2_)] were identified, with two types of bifc cations, bifc-1 and bifc-2, and four [M_2_] units, [M_2_(1)], [M_2_(2)], [M_2_(3)], and [M_2_(4)], along with one species of TCNQF_2_, all crystallographically defined (Fig. 1b and S2b†). No crystallization solvent was incorporated, providing excellent thermal stability as validated by the thermogravimetric analysis (Fig. S3†). The four CN groups of TCNQF_2_ coordinate to the axial positions of [M_2_] in a *η*^1^, *η*^1^, *η*^1^, and *η*^1^ coordination *μ*_4_ bridging mode, forming a typical fishnet layer lying on the (1−11) plane (Fig. 1c and S2c†), where four structurally distinct [M_2_] units are respectively located around TCNQF_2_ (Fig. 1a and S2a†).

The evaluation of the electronic state of each unit can be carried out by examining the local bond lengths of the unit. In the case of the [Ru_2_] units, the Ru–O_eq_ (where O_eq_ refers to the carboxylate oxygen atom of the [Ru_2_] unit) bond lengths can be used to determine the electronic state. The [Ru_2_^II,II^] and [Ru_2_^II,III^]^+^ states are generally exhibit bond lengths ranging between 2.06 to 2.07 Å and 2.02 to 2.03 Å, respectively.^55,56^ For 1, the mean Ru–O_eq_ bond lengths for the [Ru(1)_2_], [Ru(2)_2_], [Ru(3)_2_], and [Ru(4)_2_] moieties are 2.0724(10), 2.0721(9), 2.0714(9), and 2.0663(10) Å, respectively, indicating that all correspond to the [Ru_2_^II,II^] state (Table S3†).

On the other hand, it is well known that the charge state of TCNQR_*x*_ can be roughly estimated from the local bond lengths of TCNQR_*x*_ using the Kistenmacher relationship *ρ* = *A*[*c*/(*b* + *d*)] + *B*, where *b*, *c*, and *d* are the respective bond lengths for the 7,9-, 1,7-, and 1,2-positioned C–C pairs in the TCNQR_*x*_ moiety (Table S4†).^57^ Parameters *A* = −41.667 and *B* = 19.833, evaluated from TCNQ^0^ (*ρ* = 0)^58^ and Rb^+^TCNQ^−^ (*ρ* = −1),^59^ were used because of the lack of corresponding references for TCNQF_2_. The *ρ* values of 1 and 2 are −1.12(5) and −0.86(10), respectively (Table S4†), consistent with the conclusion that the TCNQF_2_ moiety adopts the monoanion radical state (TCNQF_2_˙^−^). This condition is supported by infrared (IR) spectroscopy measured at room temperature (Fig. S6a†) which showed *ν*_C≡N_ bands at 2201 cm^−1^ for 1 and 2213 and 2194 cm^−1^ for 2, assigned to the form of TCNQF_2_˙^−^ as these bands were observed at 2229 and 2219 cm^−1^ and at 2220 and 2192 cm^−1^ for TCNQF_2_ and Li^+^TCNQF_2_˙^−^, respectively. The Raman spectra of 1 and 2 were also consistent with the presence of TCNQF_2_˙^−^ (Fig. S6b†), with *ν*_C=C_ bands observed at 1407 and 1417 cm^−1^ for 1 and 2, respectively, as compared with *ν*_C=C_ bands at 1462 cm^−1^ for TCNQF_2_ and at 1426 cm^−1^ for Li^+^TCNQF_2_˙^−^.

Hence, the charge on the bifc should be +1. Indeed, the electronic state of bifc can be assessed from the Fe–Cp_cent_ distance (Cp_cent_ = center of Cp ring): the distances for neutral (fc^0^, *S* = 0) and cationic (fc^+^, *S* = 1/2) moieties are approximately 1.655 and 1.705 Å, respectively, and a valence-detrapped monocation (fc^0.5+^–fc^0.5+^) has an average distance of 1.68 Å.^45,60^ The distances in 1 and 2 are summarized in Table S5.† The average Fe–Cp_cent_ distances for bifc-1 and bifc-2 are 1.6822(11) and 1.6736(12) Å for 1 and 1.681(4) and 1.663(4) Å for 2, suggesting that both bifc moieties of bifc-1 and bifc-2 have a +1 charge; however, we cannot definitively predict the presence of the “valence-detrapped form” based solely on their structures, as the bifc moieties have inversion centers. These results indicate that the charge distribution is as shown in the formula [bifc]^+^[{M_2_^II,II^}_2_(TCNQF_2_˙^−^)] (Fig. S7†).

### Packing description of hybridized form

The hybrid layer structures of 1 and 2 are depicted as alternately stacked layers form of [D_2_A]^−^ and [bifc]^+^ molecules aligned along the [01−1] direction (Fig. 1c and S2c†). In this stacking arrangement, [Ru(1)_2_]/[Ru(3)_2_] and [Ru(2)_2_]/[Ru(4)_2_] are alternately positioned along the stacking direction (Fig. S4†), defining the interlayer translational distance (*l*_T_) corresponding to |***b***/2 − ***c***/2| (Fig. 1d and S2d†).^61,62^ The *l*_T_ values for 1 and 2 were 10.78 and 10.72 Å, respectively. The interlayer vertical distances (*l*_V_) for 1 and 2 were 9.80 and 9.71 Å, respectively, with slant angles *ϕ* (arccos(*l*_V_/*l*_T_)) of 24.6° and 25.1°, respectively (Fig. 1d and S2d†).

Two bifc molecules, bifc-1 and bifc-2, are sandwiched between the [D_2_A]^−^ layers (Fig. 1c and S2c†) located near the top and bottom sides of the TCNQF_2_ moiety (Fig. 1b, d, S2b and d†). Each bifc moiety has a distinct tilting angle *ψ*, made by the main axis of the bifc molecule, defined by the axis passing through the Cp_cent_ and D_2_A layers (Fig. 1b, d, S2b and d†). The *ψ* angles of bifc-1 and bifc-2 were 88.7° and 38.6° for 1 and 89.5° and 37.8° for 2 (*ψ* = 90° indicates a parallel orientation to the layer). While bifc-1 is positioned between the D_2_A layers, bifc-2 exhibits a tilt (Fig. 1d and S2d†). There are no π-stacking interactions involving the Cp rings of the bifc moieties, unlike 
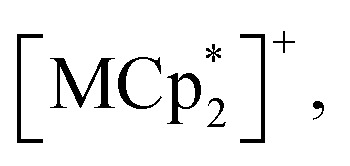
 which forms π-stacking interactions between 
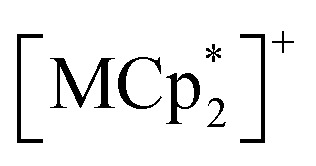
 and the TCNQ moiety of the [D_2_A]^−^ layers (referred to as π-stacked pillared layer framework; Scheme 2c).^20,43,44^ However, the formation of multiple C–H⋯F type hydrogen bonds stabilizes the alignments of the bifc cations in a densely packed form, thereby preventing the inclusion of crystallization solvents (Fig. S5 and Table S2†).^63^ The closest distances between the midpoint of TCNQF_2_ and the Fe atom of bifc-1/-2 were 4.64/5.30 Å and 4.63/5.26 Å for 1 and 2, respectively (Fig. 1b and S2b†).

### Magnetic properties of 2

**Fig. 2:**
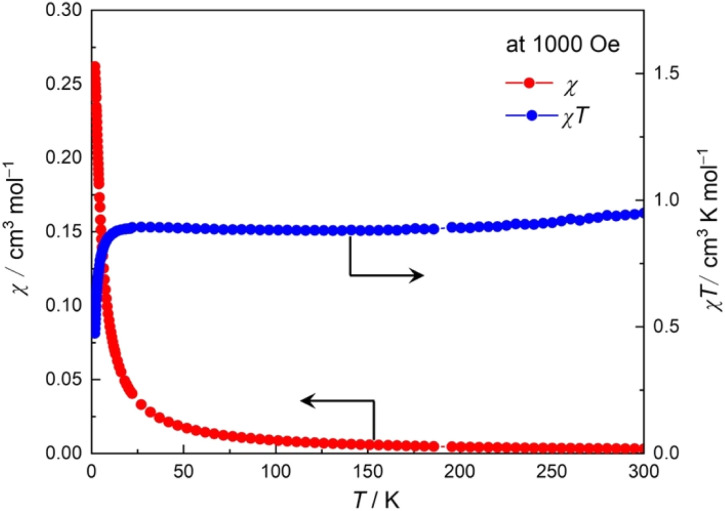
Temperature dependences of *χ* and *χT* for 2.

Because [Rh_2_] units are diamagnetic, the paramagnetic spins of the TCNQF_2_˙^−^ and [bifc]^+^ units in 2 were characterized. The temperature dependence of the magnetic susceptibility (*χ* = *M*/*H*_dc_) was measured using a direct current (DC) external field (*H*_dc_) of 1 kOe over the temperature range of 1.8 to 300 K (cooling process, Fig. 2). At 300 K, the *χT* value was 0.95 cm^3^ K mol^−1^, slightly larger than the theoretical value of 0.75 cm^3^ K mol^−1^ for the sum of two half spins of TCNQF_2_˙^−^ and [bifc]^+^ moieties with *g* = 2.0. As the temperature decreased, the *χT* value slightly decreased and stabilized at 0.89 cm^3^ K mol^−1^ at 200 K. At approximately 20 K, the *χT* value rapidly decreased to 0.47 cm^3^ K mol^−1^ at 1.8 K. The slight decrease in the *χT* value at higher temperatures could be attributed to temperature-independent paramagnetism originating from the [bifc]^+^ spin.

The rapid decrease in the *χT* value below 20 K is likely due to the effect of spin–orbit coupling of the [bifc]^+^ species^47^ and minimal intermolecular antiferromagnetic interactions.^53^ The magnetic field dependence of the magnetization at 1.8 K is shown in Fig. S8,† indicating normal paramagnetic behavior. Thus, this typical paramagnetic behavior indicates that the spins of TCNQF_2_˙^−^ and [bifc]^+^ are significantly isolated, at least in the temperature region above 20 K.

### Magnetic properties of 1

**Fig. 3:**
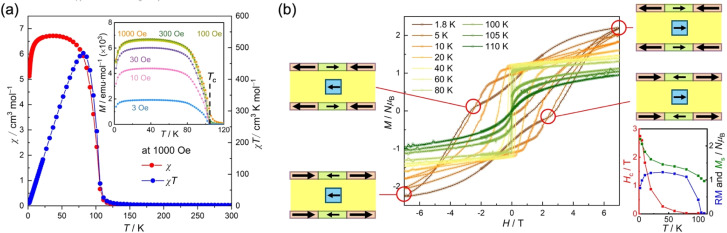
Magnetic properties of 1. (a) Temperature dependence of *χ* and *χT* measured under a 1 kOe dc field. Inset: field-cooled magnetization (FCM) measured under different external fields from 3 to 1 kOe. (b) Field dependence of magnetization at several temperatures between 1.8 and 110 K. Inset: temperature dependence of the coercive field (*H*_c_, red), the remnant magnetization (RM, blue), and the saturated magnetization at 7 T (*M*_s_, green).

**Fig. 4:**
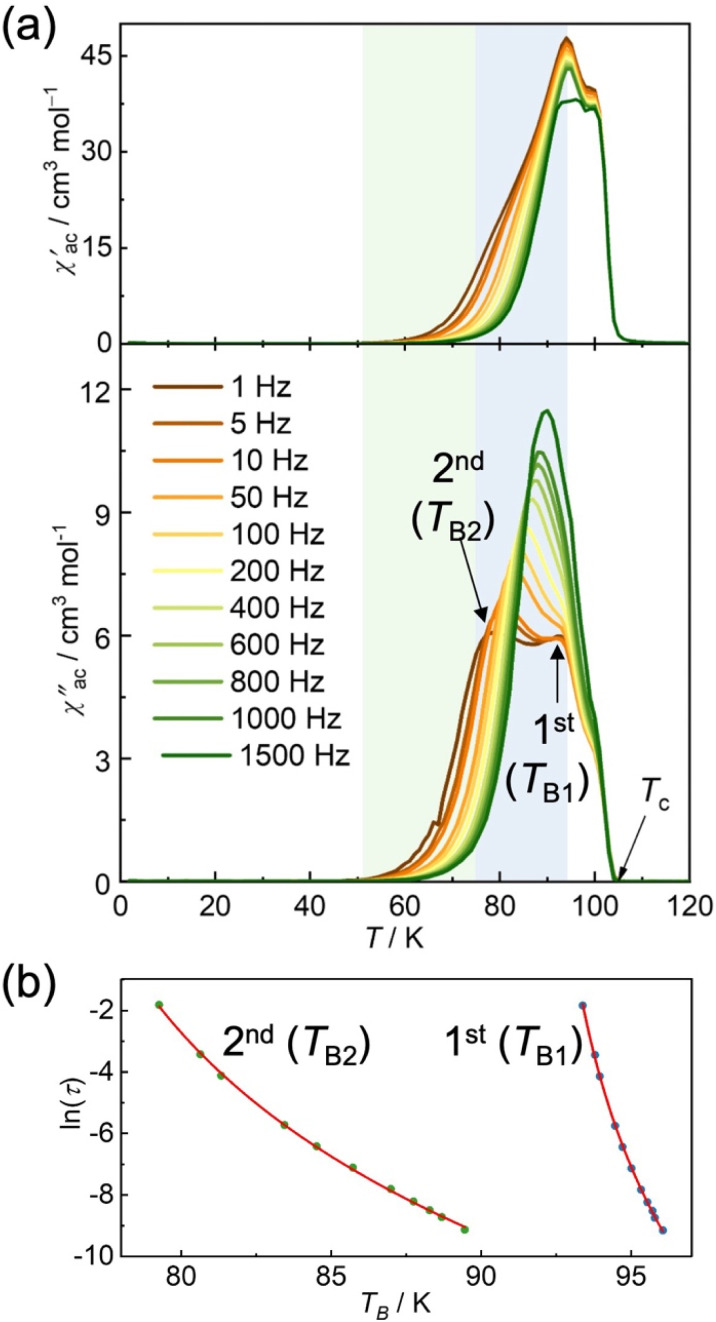
Ac susceptibilities and evaluated relaxation time as a function of temperature for 1. (a) Temperature dependence of the AC magnetic susceptibilities (*χ*′, in-phase; *χ*′′, out of phase) at zero DC field and a 3 Oe oscillating field. (b) Plots of ln(*τ*) *versus T*_B_ for 1^st^ and 2^nd^ relaxations, in which the red line represents the non-linear fitting using the critical scaling approach (see the text).

The magnetization of 1 was measured in the temperature range of 300 to 1.8 K using a field cooling method with an applied magnetic field of 1 kOe (Fig. 3a). The value of *χT* at 300 K was found to be 2.21 cm^3^ K mol^−1^, which was smaller than the theoretical value of 2.75 cm^3^ K mol^−1^ expected from individual paramagnetic contributions. This difference is attributed to the strong ferrimagnetic coupling between the spins in the D_2_A layers, with an isolated paramagnetic contribution from the [bifc]^+^ spins. As the temperature decreased, the *χT* value gradually increased and then suddenly increased at around 110 K to reach a maximum of 482.7 cm^3^ K mol^−1^ at 82 K, before rapidly dropping to 9.3 cm^3^ K mol^−1^ at 1.8 K (Fig. 3a). The *χ*–*T* curve also exhibited a sharp increase at approximately 110 K and did not decrease upon cooling until approximately 20 K, followed by a significant decrease as the temperature continued to drop to 1.8 K (Fig. 3a). Field-cooled magnetization measurements at various magnetic fields from 3 Oe to 1 kOe displayed similar behavior (inset of Fig. 3a and S9†), indicating the onset of long-range ordering at approximately 110 K. The value of *T*_c_ was finally determined to be 105 K from remnant magnetization (Fig. 3b) and alternating current (AC) susceptibility measurements (*vide infra*; Fig. 4a).

The zero-field-cooled magnetization revealed a broad peak in the temperature range of 40 to 100 K at low applied fields, suggesting the occurrence of spin reorientation or fractural long-range magnetic ordering (Fig. S9†). The decrease in magnetization at low temperatures below 20 K is attributed to the magnetic anisotropy of the [bifc]^+^ spins due to spin–orbit coupling,^47,53^ consistent with observations in similar compounds containing the 
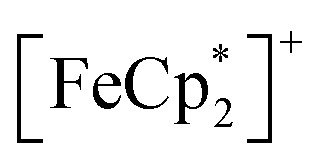
 unit.^20^

The characteristic behavior of the hybrid structure containing [bifc]^+^ spins can also be observed in the field dependence of magnetization (*M*–*H* curves; Fig. 3b), which exhibits significant hysteresis akin to 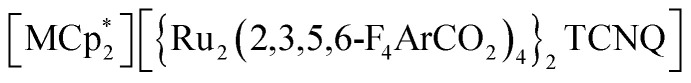 (M = Fe, Cr).^20,43^ However, it differs from the typical D_2_A series of [Ru_2_]_2_TCNQR_*x*_^61,62^ compounds by displaying “gourd-shaped” features at low temperatures below 20 K. The coercive field decreased quasi-exponentially with increasing temperature and eventually disappeared at approximately 100 K, corresponding to *T*_c_. Below 20 K, the remnant magnetization (RM) in the *χ*–*T* curve shows a declining trend, attributed to the magnetic anisotropy of [bifc]^+^ (insert of Fig. 3b). The “gourd-shaped” hysteresis feature observed at temperatures below 20 K is associated with the gradual flipping of anisotropic [bifc]^+^ transitioning from parallel alignment at high fields to antiparallel alignment at approximately ±3 T relative to the magnetic moment of the layer (Fig. 3b).

The temperature dependence of the AC magnetic susceptibilities (*χ*′: in-phase, *χ*′′: out-of-phase) was measured under a zero DC field and an oscillating field of 3 Oe in the frequency range of 1 to 1.5 kHz (Fig. 4a). Upon cooling from 120 K, the *χ*′ value exhibited a sudden increase at approximately 105 K, forming a distinct frequency-independent cusp at 100 K. As the temperature decreased further, a second peak appeared at approximately 94 K, followed by a broad tailing in the temperature range (∼50 K) that showed frequency dependence. The behavior of *χ*′′ provides insight into the nature of the long-range ordering. At 105 K, the *χ*′′ deviated from the baseline to form a shoulder at approximately 100 K with no discernible frequency dependence, indicating the onset of long-range ferrimagnetic ordering at *T*_c_ = 105 K (consistent with the RM data). Upon further cooling, two frequency-dependent peaks were observed at approximately 93 and 77 K at 1 Hz, which shifted to higher temperatures with increasing frequency. The frequency-dependent peaks at high (*T*_B1_) and low (*T*_B2_) temperatures are referred to as the 1^st^ and 2^nd^ relaxation processes, respectively (Fig. 4a). The behavior of *χ*′′ shows the characteristic properties of a hybridized layered magnet composed of well-ordered ferrimagnetic layers [D_2_A]^−^ and sandwiched paramagnetic [bifc]^+^ species (Fig. 4a). It should be noted that some layered magnets with interstitial crystallization solvents exhibit similar frequency-dependent AC susceptibility behavior with tailing over a wide temperature range. This could be owing to the presence of defects resulting from the partial elimination of interstitial solvents, which produce different magnetic domains.^61,62,64,65^ However, the behavior of *χ*′′ in 1 was expected to be different because it remains stable even in the absence of a crystallization solvent. Although the origins of the three peaks at *T*_c_, *T*_B1_, and *T*_B2_ (where *T*_B1_ and *T*_B2_ are frequency-dependent) may seem complicated, they can fundamentally be attributed to the individually related magnetic ordering of [D_2_A]^−^ layers and [bifc]^+^ species. The long-range ordering at *T*_c_ likely results from ferrimagnetic ordering associated with interlayer ferromagnetic dipole interactions between the ferrimagnetically ordered [D_2_A]^−^ layers. The strong magnetic coupling within the [D_2_A]^−^ layer facilitates magnetic long-range ordering involving interlayer ferromagnetic dipole interactions, which consequently neglects sandwiched paramagnetic spins, as observed in other 
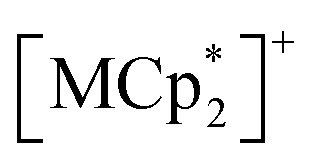
 sandwiched layer systems.^20,43^ Additionally, the nature of ferrimagnetic ordering agrees with the empirical rule for assigning interlayer magnetic interaction, whether ferromagnetic or antiferromagnetic, using the interlayer translational distance (*l*_T_). The value of *l*_T_ = 10.78 Å for 1 falls within the range for ferromagnetic domination (>10.3 Å),^61,62^ resulting in a bulk ferrimagnet.

The presence of two types of frequency-dependent peaks at *T*_B1_ and *T*_B2_ can be attributed to magnetic long-range ordering, which is influenced by the spins of the [bifc]^+^ cations. These dynamic peaks may result from two distinct orientations for the [bifc]^+^ cations, influenced by their strong magnetic anisotropy.^51,66^ The relaxation time, *τ*, at each *T*_B_ was determined from the peak of *χ*′′ and Fig. 4b displays ln(*τ*) *vs. T* plots for the first and second relaxation processes, revealing non-linear curves. Notably, the isolated [bifc]^+^ spins observed in Fig. 2 for 2 do not exhibit any anomalies in this temperature range. Consequently, these relaxations are thought to be associated with glassy behavior involving the subsequent ordering of the [bifc]^+^ spins, influenced by the internal dipole fields produced by long-range ordering related to the interlayer interactions *via J*_NNNI_ at *T*_c_.

**Fig. 5:**
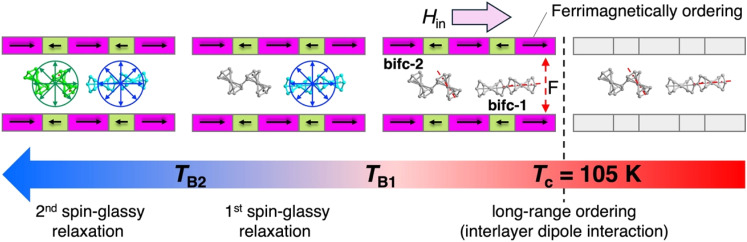
Schematic representation of stepwise spin ordering processes in 1 upon cooling (from right to left), where F represents ferromagnetic interaction; gray color components mean their indicate paramagnetic behavior; and the purple and green units represent [Ru_2_^II,II^] with *S* = 1 and TCNQF_2_˙^−^ with *S* = 1/2, respectively, which ferrimagnetically order to produce *H*_in_ following in the direction parallel to the layer parallel direction.

To investigate the relaxation mechanism, we analyzed the dynamic behavior using a critical scaling approach (ESI†): *τ* = *τ*_0_(*T*_B_/*T*_SG_ − 1)^−*zv*^, where *τ* = 1/(2π*f*) represents the relaxation time at a frequency *f*, *τ*_0_ is the characteristic relaxation time of the system, *T*_SG_ is the spin glass transition temperature as *f* is extrapolated to zero, *zv* is the dynamic critical exponent, and *T*_B_ is the blocking temperature, which is defined as the peak temperature in *χ*′′−*T* plots.^67,68^ The best-fit result are shown in Fig. 4b. The obtained parameter sets for the 1^st^ and 2^nd^ relaxations are *τ*_0_ = 2.6 × 10^−14^ s, *zv* = 7.1, and *T*_SG_ = 91.9 K, and *τ*_0_ = 2.7 × 10^−9^ s, *zv* = 6.9, and *T*_SG_ = 73.6 K, respectively. The value of *zv* falls within the typical range for spin-glass systems, which is between 4 and 12.^69^ The significantly faster *τ*_0_ times compared to the intrinsic cluster spin relaxation times, ranging from 1 × 10^−6^ to 1 × 10^−7^ s, are consistent with the behavior observed in glassy systems, including spin glasses.^69^ Although the characteristic anisotropic magnetic behavior of this type of metallocene compound is typically observed at low temperatures (*T* < 20 K),^70^ the *H*_in_ generated by the established magnetization of the D_2_A layer kinetically suppresses the thermal motion of the [bifc]^+^ spins, causing them to freeze below approximately 95 K (= *T*_B1_) (Fig. 5).

This two-step relaxation process can be attributed to two types of [bifc]^+^ radicals with distinct packing modes (Fig. 5). The magnetic easy axis of [bifc]^+^ is along the Fe–Cp_cent_ axis, as shown in Fig. 1b. Notably, the magnetization easy axis of the two-dimensional [Ru_2_]_2_TCNQR_*x*_ system should be parallel to the magnetic moment of the [D_2_A]^−^ layer (see ESI, Fig. S11†), implying that the direction of *H*_in_ is parallel to the layer. Because the anisotropic easy axis of bifc-1 (*ψ* = 88.7°) is almost parallel to the direction of *H*_in_, whereas that of bifc-2 (*ψ* = 38.6°) is tilted (Fig. 1b and d), the spin motion of bifc-1 is more easily inhibited by *H*_in_ than that of bifc-2 (Fig. 5). Consequently, the 1^st^ relaxation at high temperatures originates from bifc-1 and the 2^nd^ from bifc-2. Interestingly, the two relaxation peaks appeared to merge under an external field of 60 Oe (Fig. S12†).

The value of the internal magnetic field was estimated using mean-field approximation. The internal magnetic field *H*_m_ at 0 K of a ferromagnetic material can be expressed as *H*_m_ = 3*S*_i_*k*_B_*T*_c_/{(*S*_i_ + 1)*M*_s_}, where *S*_i_ is the spin quantum number, *k*_B_ is the Boltzmann constant, *T*_c_ is the magnetic phase-transition temperature, and *M*_s_ is the saturation magnetization.^71^ The present system is a ferrimagnet with anisotropic low-dimensionality; therefore, this estimation may not be appropriate for this case. However, for simplicity, the total spin number *S*_T_ = 3/2 (=1 + 1 − 1/2) for the [D_2_A]^−^ layer was used as *S*_i_. Using *M*_s_ ≈ 2.2 *μ*_B_, which is the magnetization under 7 T at 1.8 K, and *T*_c_ = 105 K, the molecular magnetic field can be estimated to be 1.27 × 10^2^ T (1.01 × 10^8^ A m^−1^). In this approximation, *H*_m_ was directly proportional to *T*_c_. Therefore, *H*_m_ is approximately an order of magnitude smaller than that of Fe and Ni.^71^ However, it still suggests that a large internal magnetic field of over 100 T is present in this case.

It should be noted that the *T*_c_ value of 1 exceeds those reported for previously known hybrid D/A-MOF magnets based on [Ru_2_] and TCNQR_*x*_, most of which were below 100 K.^20,43^ The structural assessment demonstrates that the alignment of the donor and acceptor is more linear in 1 compared to previously reported π-stacked pillared layer frameworks (π-PLFs), enhancing the orbital overlap between the π* of TCNQR_*x*_˙^−^ and [Ru_2_] in 1. The π* (TCNQR_*x*_˙^−^)–π* ([Ru_2_]) overlap can be assessed by the π part angular overlap model, expressed as *A*_p_ = {1 − (sin *δ* sin *ω*)^2^}^0.5^ (see ESI, Table S6†).^72^ Compared with other π-PLFs, *A*_p_ of 1 is close to 1, indicating a larger overlap and charge transfer interaction, resulting in a higher *T*_c_. The linear arrangement of [Ru_2_] and TCNQF_2_ in 1 may have been favored because of the sterically bulky [bifc]^+^.

## Conclusions

Two magnetic hybrids, [bifc][{M_2_(2,3,5,6-F_4_ArCO_2_)_4_}_2_(TCNQF_2_)] (M = Ru, 1; M = Rh, 2), were synthesized. Compounds 1 and 2 underwent electron transfer from [bifc] to TCNQF_2_, resulting in a [bifc]^+^[(M_2_^II,II^)_2_(TCNQF_2_˙^−^)] electronic state. Compound 2 exhibited paramagnetic behavior, whereas 1 displayed long-range ferrimagnetic ordering and unprecedented two-step relaxation dynamics. A superior transition into a ferrimagnet at *T*_c_ = 105 K was achieved by ferromagnetically coupling the ferrimagnetic layers of [{Ru_2_(2,3,5,6-F_4_ArCO_2_)_4_}_2_(TCNQF_2_)]^−^ to generate an effective interlayer magnetic field, *H*_in_. Stepwise dynamic ordering with spin-glassy features was observed below 95 K, where the two types of oriented [bifc]^+^ spins (*S* = 1/2) were individually influenced by *H*_in_. Such stepwise magnetic ordering may only be possible in cases where (1) the intralayer magnetic ordering (*i.e.*, short-range ordering) is sufficiently strong to produce a large magnetic moment at such a high temperature (100 K); and (2) the interlayer dipole interaction *J*_NNNI_ is stronger than the interactions between the encapsulated paramagnetic spin and the magnetic layer *J*_NNI_, that is, *J*_NNNI_ > *J*_NNI_. Given the scenario where *J*_NNNI_ < 0 (indicating an antiferromagnetic interaction), a spin-frustration state is anticipated when the hybrid layers are magnetically ordered. This study introduces a novel approach for constructing ternary hybrid magnets using Z^+^[D_2_A]^−^ systems comprising functional molecules (anisotropic paramagnetic Z^+^) and low-dimensional molecule-based magnets [D_2_A]^−^. These hybrid magnets facilitate the observation of the dynamic ordering of intercalated paramagnetic spins influenced by *H*_in_. It is important to note that this type of hybrid material provides a valuable platform for investigating the nature of magnetic ordering in low-dimensional materials and has the potential to serve as a platform for investigating unique phenomena that occur between functional guests and magnetic hosts that possibly generate a strong *H*_in_.

## Data availability

All data supporting the findings of this study, including details of the experimental study, are available in the article and ESI.†

## Author contributions

H. M. conceived the study. Q. L. prepared and characterized the materials. W. K. and H. M. supervised the experiments. Q. L., W. K., and H. M. prepared the manuscript.

## Conflicts of interest

There are no conflicts to declare.

## Supplementary Material

SC-OLF-D4SC04722B-s001

SC-OLF-D4SC04722B-s002
