# Supplementary material for: Dynamic spin reordering in a hybrid layered ferrimagnet with intercalated biferrocenium radicals
Source: Chem Sci. 2024 Oct 30;15(46):19411–9. doi: 10.1039/d4sc04722b (PMC11537288; doi:10.1039/d4sc04722b)
Supplement: SC-015-D4SC04722B-s001 [file SC-015-D4SC04722B-s001.pdf]

Supplementary Information (SI) for Chemical Science.
This journal is © The Royal Society of Chemistry 2024

Supporting Information

Dynamical Spin Reordering in a Hybrid Layered Ferrimagnet with Intercalated Biferrocenium Radicals

Qingxin Liu,[a,b] Wataru Kosaka,[a,b] Hitoshi Miyasaka*[a,b]

[a] Institute for Materials Research, Tohoku University, 2-1-1 Katahira, Aoba-ku, Sendai 980-8577, Japan

[b] Department of Chemistry, Graduate School of Science, Tohoku University, 6-3 Arama-ki-Aza-Aoba, Aoba-ku, Sendai 980-8578, Japan

Corresponding author*

Prof. Dr. Hitoshi Miyasaka

Institute for Materials Research

Tohoku University

2–1–1 Katahira, Aoba-ku, Sendai 980-8577, Japan

E-mail: miyasaka@imr.tohoku.ac.jp

E-mail: hitoshi.miyasaka.e7@tohoku.ac.jp

Tel: +81-22-215-2030

FAX: +81-22-215-2031

Contents for SI

Contents for SI ··· S2
Experimental Section ··· S3
Table S1. Crystallographic data ··· S5
Table S2. Bond distances and angles of hydrogen bond ··· S6
Table S3. Selected bond lengths around metal centers ··· S7
The oxidation state of the [Ru_2] unit in 1 ··· S7
Table S4. Bond distances in the TCNQ moiety ··· S8
Table S5. Fe-Cp_{center} distances ··· S9
Table S6. The estimation of overlap integral ··· S10
Fig. S1 PXRD patterns ··· S11
Fig. S2 Structure of **2** ··· S12
Fig. S3 The TGA curves ··· S13
Fig. S4 Packing views projected perpendicular to the (1−11) plane ··· S14
Fig. S5 Packing diagrams representing C−H···F type hydrogen bond ··· S15
Fig. S6 Infrared and Raman spectra ··· S17
Fig. S7 Electronic and spin states of assembly units ··· S18
Fig. S8 Magnetic field dependence of magnetization at 1.8 K of **2** ··· S19
Fig. S9 FCM, ZFCM and RM curves of **1** at several field ··· S20
Relaxation time analysis of spin glass behavior ··· S21
Fig. S10 Multiple peak fit results of χ'' of **1** with Gauss function ··· S22
Table S7 χ'' peak positions of fit peaks after separation ··· S23
Stepwise relaxation behavior in 1 ··· S24
Fig. S11 Spin alignments in D_2A-type 2D antiferromagnetic phase ··· S24
Fig. S12 Temperature dependence of χ'' of **1** under DC fileds ··· S25
Reference ··· S26

Experimental Section

General procedures and materials.

All synthetic procedures were performed under an inert atmosphere using standard Schlenk-line techniques and a commercial glove box. Chemicals were of reagent grade and purchased from commercial sources. Solvents were dried using common drying agents and distilled under nitrogen before use. The starting materials $TCNQF_2$ [1] and $[Ru/Rh_2^{II,II}(2,3,5,6\text{-}F_4ArCO_2)_4(THF)_2]$ [2,3] and $bifc^+I_3^-$ [4] were prepared following previously reported methods. Biferrocene was synthesized from bromoferrocene via Ullmann reactions[5] and further purified by column chromatography using *n*-hexane as the eluent.[6]

Synthesis of (bifc)[{Ru_2(2,3,5,6-F_4ArCO_2)$_4$}$_2$($TCNQF_2$)] (1). A solution containing TCNQF2 (2.40 mg, 0.01 mmol) and biferrocene (3.70 mg, 0.01 mmol) in dichloromethane (DCM) (20 mL) was prepared and carefully layered as a bottom layer in narrow-diameter glass tubes (inner diameter of 8 mm). Separately, $[Ru_2(2,3,5,6\text{-}F_4ArCO_2)_4(THF)_2]$ (22.4 mg, 0.02 mmol) dissolved in *p*-xylene (20 mL) was added in 2 mL portions onto the prepared bottom layer in each glass tube. The tubes were sealed and left undisturbed for one week to allow the crystallization of block-shaped black crystals of **1** (yield: 28%). Thermogravimetric analysis indicated that the crystals lost a small amount of surface solvent, but no solvent of crystallization was observed. Elemental analysis (calc.: C, 41.30; H, 1.10; N, 2.19%; found: C, 41.32; H, 1.28; N, 2.34%). IR (KBr): $\nu_{(C\equiv N)}$ 2201 cm^{-1}.

Synthesis of (bifc)[{Rh_2(2,3,5,6-F_4ArCO_2)$_4$}$_2$($TCNQF_2$)] (2). The synthesis of the crystalline sample of **2** for SCXRD analysis was analogous to that of **1**, with the exception that $[Ru_2(2,3,5,6\text{-}F_4ArCO_2)_4](THF)_2$ was mixed with $[Rh_2(2,3,5,6\text{-}F_4ArCO_2)_4](THF)_2$ and *p*-xylene was mixed with DCE (yield: 22%). Elemental analysis (calc.: C, 41.18; H, 1.10; N, 2.18%; found: C, 41.31; H, 1.23; N, 2.35%). IR (KBr): $\nu_{(C\equiv N)}$ 2213 and 2194 cm^{-1}.

Physical characterization.

IR spectra were recorded on a KBr pellet using a Jasco FT-IR 4200 spectrophotometer. Thermogravimetric analyses were conducted on a Shimadzu DTG-60H apparatus under an N_2 atmosphere in the temperature range from room temperature to 400 °C at a heating rate of 5 °C min^{-1}. The powder XRD patterns were collected using a Rigaku Ultima IV diffractometer equipped with Cu Kα radiation (λ = 1.5418 Å). Powder reflection spectra were measured on pellets diluted with $BaSO_4$ using a Shimadzu UV-3150 spectrometer. Magnetic susceptibility measurements were performed with a Quantum Design SQUID magnetometer (MPMS-XL) in the temperature ranges of 1.8 to 300 K and in DC magnetic fields from –7 to 7 T. Polycrystalline samples embedded in liquid paraffin were also analyzed. The experimental data were corrected for contributions from the sample holder, liquid paraffin, and diamagnetic effects calculated using Pascal constants.[7]

Crystallography.

Crystal data for **1** and **2** were collected using a CCD diffractometer (Rigaku Saturn724M+HyPix-6000HE) with multilayer mirror monochromated Mo Kα radiation (λ = 0.71073 Å). Single crystals with dimensions of 0.08 × 0.09 × 0.1 mm and 0.02 × 0.02 × 0.025 mm for **1** and **2**, respectively, were mounted on a thin Kapton film using Nujol and cooled to 102 K in an N_2 gas stream. Using Olex2,[8] the crystal structures were solved with the SHELXS[9] structure solution program using Direct Methods and refined using the SHELXL[10] refinement package through least-squares minimization. Anisotropic refinement of non-hydrogen atoms was performed using the full-matrix least-squares method on F^2. Non-hydrogen atoms were hydrogenated based on the theoretical background of structural chemistry, and hydrogen atoms were fixed in their positions on the carbon atoms. The crystallographic data, parameters for data collection, and specifics of the structure refinement are detailed in Table S1. These datasets have been deposited as CIFs at the Cambridge Data Centre as supplementary publication notes. CCDC 2371166 and 2371167 for **1** and **2**, respectively. Structural diagrams were generated using the Diamond software.[11]

Table S1. Crystallographic data for **1** and **2**.

Compounds	**1**	**2**
Empirical formula	$C_{88}H_{28}F_{34}Fe_2N_4O_{16}Ru_4$	$C_{88}H_{28}F_{34}Fe_2N_4O_{16}Rh_4$
Formula weight	2559.12	2566.48
Temperature / K	102.1	102.1
Crystal system	triclinic	triclinic
Space group	$P\bar{1}$	$P\bar{1}$
a / Å	15.6986(3)	15.6132(4)
b / Å	17.2101(3)	17.0211(4)
c / Å	17.6280(3)	17.6801(5)
α / °	76.436(2)	76.270(2)
β / °	71.465(2)	71.301(2)
γ / °	89.910(2)	89.251(2)
Volume / Å^3	4375.87(15)	4313.2(2)
Z	2	2
ρ_{calc} / g·cm^{-3}	1.942	1.976
μ / mm^{-1}	1.139	1.220
F_{000}	2492.0	2500.0
Crystal size / mm^3	0.10 × 0.09 × 0.08	0.03 × 0.02 × 0.02
Radiation	Mo $K\alpha$ (λ = 0.71073)	Mo $K\alpha$ (λ = 0.71073)
2θ range for data collection / °	3.042 to 59.444	3.056 to 51.362
Index ranges	$-21 \leq h \leq 21, -23 \leq k \leq 22, -24 \leq l \leq 21$	$-19 \leq h \leq 19, -20 \leq k \leq 20, -21 \leq l \leq 21$
Reflections collected	54325	46734
Independent reflections	20837 [R_{int} = 0.0518]	16279 [R_{int} = 0.0658]
Data/restraints/parameters	20837/12/1333	16279/18/1333
Goodness-of-fit on F^2	1.025	1.026
Final R^{1-2} indexes [I>=2σ (I)]	R_1 = 0.0394, wR_2 = 0.0865	R_1 = 0.0732, wR_2 = 0.1660
Final R^2 indexes [all data]	R_1 = 0.0603, wR_2 = 0.0923	R_1 = 0.1246, wR_2 = 0.1870
CCDC No.	2371166	2371167

$R_1 = \sum||F_c| - |F_o|| / \sum|F_o|; wR_2 = [\sum w(F_o^2 - F_c^2)^2/\sum w(F_o^2)^2]^{1/2}$

Table S2. Bond distances (Å) and angles (°) of C−H···F−C hydrogen bond.

C−H	F	H···F distance	C−H···F angle	C−H	F	H···F distance	C−H···F angle
**1**							
	bifc-1				bifc-2		
C69−H69	$F33^{i}$	2.541	168.1	C79−H79	$F21^{iii}$	2.562	125.1
C70−H70	$F28^{i}$	2.763	130.9	C80−H80	$F20^{iii}$	2.742	131.7
	$F9^{ii}$	2.800	116.9		F26	2.880	162.8
	$F10^{ii}$	2.870	168.9		F25	2.874	109.9
	$F27^{i}$	3.034	117.5	C81−H81	$F32^{iv}$	2.919	118.6
C71−H71	$F9^{ii}$	2.826	115.8		F25	2.714	115.7
C72−H72	F33	3.094	158.0	C82−H82	$F31^{iv}$	2.402	147.1
C74−H74	$F27^{i}$	2.723	134.8	C83−H83	$F34^{iii}$	2.356	151.8
C75−H75	F13	3.033	108.9	C85−H85	F25	3.085	117.0
C76−H76	F13	2.688	123.23	C86−H86	$F11^{iii}$	2.551	147.3
	F14	2.750	115.1		$F12^{iii}$	2.856	152.6
	F34	3.009	173.4	C88−H88	F12	2.918	150.2
C77−H77	F29	2.686	126.2				
C78−H78	F29	3.055	111.4				
	F30	2.529	166.6				
**2**							
	bifc-1				bifc-2		
C69−H69	$F33^{i}$	2.554	170.4	C79−H79	$F21^{iii}$	2.575	126.9
C70−H70	$F28^{i}$	2.752	131.7	C80−H80	$F20^{iii}$	2.750	130.6
	$F9^{ii}$	2.832	116.7		F26	2.809	162.6
	$F10^{ii}$	2.845	167.6		F25	2.915	109.2
	$F27^{i}$	3.143	115.9	C81−H81	$F32^{iv}$	2.868	120.5
C71−H71	$F9^{ii}$	2.818	117.0		F25	2.727	115.8
C72−H72	F33	3.029	159.9	C82−H82	$F31^{iv}$	2.402	147.1
C74−H74	$F27^{i}$	2.639	136.3	C83−H83	$F34^{iii}$	2.325	149.3
C75−H75	F13	3.0729	108.7	C85−H85	F25	2.988	119.4
C76−H76	F13	2.732	123.7	C86−H86	$F11^{iii}$	2.520	148.9
	F14	2.724	116.4		$F12^{iii}$	2.837	150.2
	F34	3.027	174.3	C88−H88	F12	2.907	148.9
C77−H77	F29	2.710	124.1				
C78−H78	F29	2.957	114.9				
	F30	2.538	170.5				

The symmetry operations: (i) $-x + 1, -y, -z + 2$, (ii) $-x + 1, -y, -z + 1$, (iii) $-x + 1, -y + 1, -z + 1$, (iv) $-x + 1, -y + 1, -z + 2$.

Table S3. Selected bond lengths (Å) around metal centers for **1** and **2**.

	**1**	**2**
M1–O1	2.0629(19)	2.033(5)
M1–O2^{a}	2.0781(19)	2.048(5)
M1–O3	2.0714(19)	2.035(6)
M1–O4^{a}	2.0770(19)	2.045(5)
M2–O5	2.069(2)	2.033(5)
M2–O6^{b}	2.072(2)	2.044(6)
M2–O7	2.0687(18)	2.043(4)
M2–O8^{b}	2.0780(18)	2.037(4)
M3–O9	2.0657(18)	2.042(5)
M3–O10^{c}	2.0727(3)	2.028(5)
M3–O11	2.0724(18)	2.030(5)
M3–O12^{c}	2.0749(18)	2.033(5)
M4–O13	2.0766(19)	2.040(4)
M4–O14^{d}	2.0603(19)	2.034(4)
M4–O15	2.0676(19)	2.029(5)
M4–O16^{d}	2.0606(19)	2.021(5)
M1–N1	2.289(2)	2.213(5)
M2–N2	2.252(2)	2.188(6)
M3–N3	2.265(2)	2.192(5)
M4–N4	2.274(2)	2.212(6)
M1–M1^{a}	2.2904(4)	2.4063(10)
M2–M2^{b}	2.2817(4)	2.3940(10)
M3–M3^{c}	2.2913(4)	2.4027(9)
M4–M4^{d}	2.2897(4)	2.4041(10)

Symmetry codes: ^{a} $-x, -y, -z + 2$, ^{b} $-x + 1, -y, -z + 1$, ^{c} $-x + 2, -y + 1, -z + 1$, ^{d} $-x + 1, -y + 1, -z + 2$.

The oxidation state of the [Ru$_2$] unit in 1. The oxidation state of [Ru$_2$] unit can be known from the Ru–O$_{eq}$ length (O$_{eq}$ = equatorial oxygen atoms), which is quite sensitive to the oxidation state of the [Ru$_2$] unit and to be 2.06–2.07 Å for [Ru$_2^{II,II}$] and 2.02–2.03 Å for [Ru$_2^{II,III}$]$^+$.[12,13] The average Ru–O$_{eq}$ length of **1** is 2.0724(10), 2.0721(9), 2.0714(9). and 2.0663(10) Å for [Ru(1)$_2$], [Ru(2)$_2$], [Ru(3)$_2$] and [Ru(4)$_2$] unit, respectively, indicating that the all [Ru$_2$] units in **1** is [Ru$_2^{II,II}$].

Table S4. Bond distances in the TCNQ moiety and degree of charge transfer (ρ) estimated from the Kistenmacher relationship.[14]

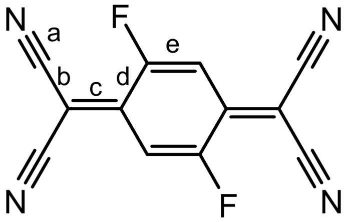

Compound	charge	*a*	*b*	*c*	*d*	*e*	ρ
TCNQ[15]	0	1.140(1)	1.441(1)	1.374(3)	1.448(4)	1.346(3)	0 (fix)
RbTCNQ[16]	−1	1.153(7)	1.416(8)	1.420(1)	1.423(3)	1.373(1)	−1 (fix)
		1.149(3)	1.418(4)	1.424(4)	1.411(4)	1.363(4)	
		1.148(3)	1.412(4)		1.418(4)		
**1**		1.150(3)	1.420(4)	1.426(4)	1.418(3)	1.352(4)	−1.12(5)
		1.143(3)	1.421(4)		1.414(4)		
		Av. 1.1475(15)	Av. 1.418(2)	Av. 1.425(3)	Av. 1.4157(18)	Av. 1.358(3)	
		1.135(8)	1.414(9)	1.412(9)	1.427(9)	1.354(9)	
		1.127(8)	1.426(9)		1.412(9)		
**2**		1.141(8)	1.425(9)	1.411(8)	1.430(9)	1.348(9)	−0.86(10)
		1.130(8)	1.420(9)		1.415(9)		
		Av 1.133(4)	Av 1.421(5)	Av 1.411(6)	Av 1.421(5)	Av 1.351(6)	

Table S5. Fe-Cp_{center} distances and the electronic states of biferrocene in **1** and **2**.

Compounds	Fe site	Fe-Cp_{center} / Å	Electronic state
**1**	Fe1	1.6698(16)<br>1.6946(16)<br>Av. 1.6822(11)	$Bifc^{+1}$<br>$(Fe^{II}–Fe^{III})^{+}$<br>valence-detrapped
	Fe2	1.6820(16)<br>1.6629(18)<br>Av. 1.6736(12)	
**2**	Fe1	1.674(5)<br>1.692(6)<br>Av 1.681(4)	$Bifc^{+1}$<br>$(Fe^{II}–Fe^{III})^{+}$<br>valence-detrapped
	Fe2	1.671(5)<br>1.655(5)<br>Av 1.663(4)	

Table S6. The estimation of the overlap integral.

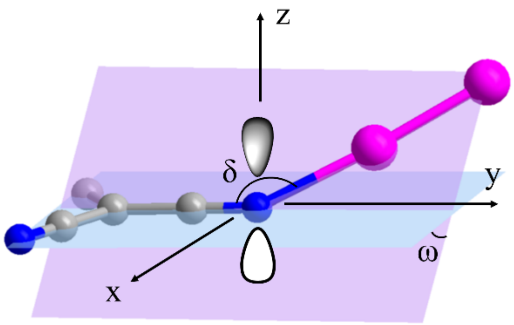

Compounds	Asymmetric unit	δ / ° [a]	ω / ° [b]	A_σ [c]	A_π [d]	T_c / K
	Ru1	149.3	38.0	0.0991	0.9492	
**Ref. 1**[e]	Ru2	164.0	18.1	0.0073	0.9963	82
	average	156.6	28.1	0.0348	0.9824	
	Ru1	152.0	37.4	0.0814	0.9584	
**Ref. 2**[e]	Ru2	165.4	17.3	0.0056	0.9972	88
	average	158.7	27.3	0.0278	0.9860	
	Ru1	149.8	37.2	0.0929	0.9524	
**Ref. 3**[e]	Ru2	163.0	18.2	0.0083	0.9958	89
	average	156.4	27.7	0.0347	0.9825	
	Ru1	157.6	4.6	0.0009	0.9995	
	Ru2	156.3	20.4	0.0197	0.9901	
**1**	Ru3	152.5	13.8	0.0122	0.9939	105
	Ru4	151.9	7.7	0.0040	0.9980	
	average	154.6	11.6	0.0075	0.9965	

[a] ∠Ru-N-C; [b] Dihedral angle between two least-square plane formed by (Ru−Ru−N≡C−C) and (C−C−(C≡N)$_2$); [c] $A_\sigma = (sin\delta \cdot sin\omega)^2$;[17] [d] $A_\pi = \{1 - (sin\delta \cdot sin\omega)^2\}^{0.5}$;[6] [e] **Ref. 1-3** are reported compound (FeCp*$_2$)[$Ru_2^{II,II}$(2,3,5,6-F_4ArCO_2)$_4$]$_2$(TCNQ)·2DCE, (CoCp*$_2$)[$Ru_2^{II,II}$(2,3,5,6-F_4ArCO_2)$_4$]$_2$(TCNQ)·2DCE and (CrCp*$_2$)[$Ru_2^{II,II}$(2,3,5,6-F_4ArCO_2)$_4$]$_2$(TCNQ)·2DCE, respectively.[18,19]

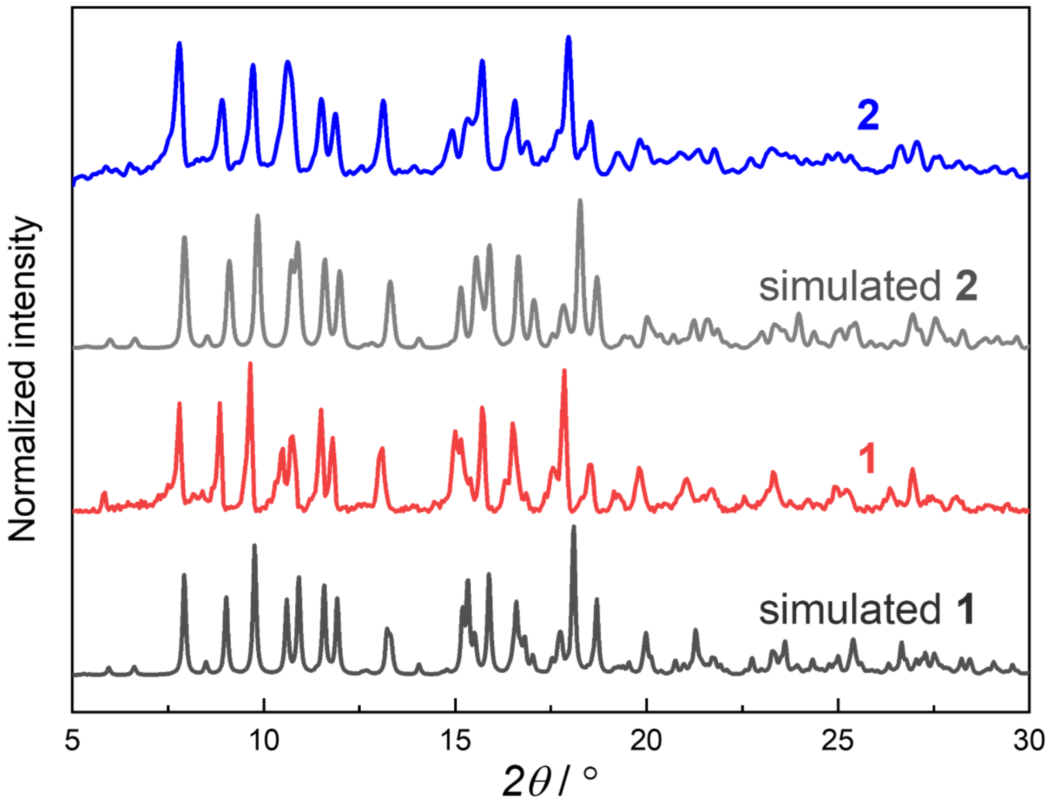

Figure S1. Experimental (colored) and simulated (black and gray) PXRD patterns of **1** and **2** at room temperature.

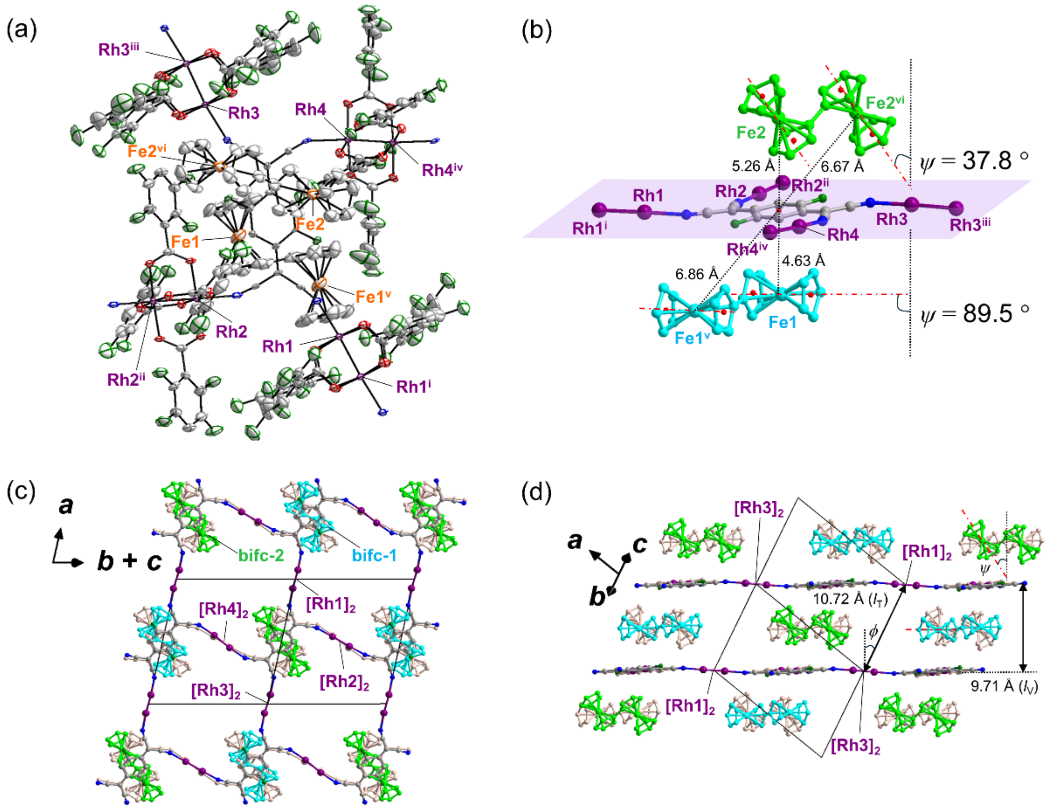

Figure S2. Structure of **2**. (a) Thermal ellipsoid plots (using 50% probability ellipsoids) of the asymmetric unit, in which hydrogen atoms are omitted for clarity, and O, C, N, F, Fe, and Rh atoms are represented in red, gray, blue, green, orange, and violet, respectively. The symmetry operations are (i) $-x$, $-y$, $-z + 2$, (ii) $-x + 1$, $-y$, $-z + 1$, (iii) $-x + 2$, $-y + 1$, $-z + 1$, (iv) $-x + 1$, $-y + 1$, $-z + 2$, (v) $-x + 1$, $-y$, $-z + 2$, (vi) $1-x$, $1-y$, $1-z$. (b) Packing diagrams focused on the location of bifc. Packing views along [01−1] (c) and [011] (d) direction. Hydrogen atoms and 2,3,5,6-$F_4ArCO_2^-$ ligands around the Rh centers are omitted for clarity. Only the forefront subunits are depicted in color, while others are colored in pale brown. The atomic colors are the same as those in panel (a) except for bifc; bifc-1 and bifc-2 are colored in cyan and yellow-green, respectively. The red dash-dot line in panel (b) and (d) represents the main axis of bifc. The purple plane in panel (b) represents the (1−11) D_2A layer.

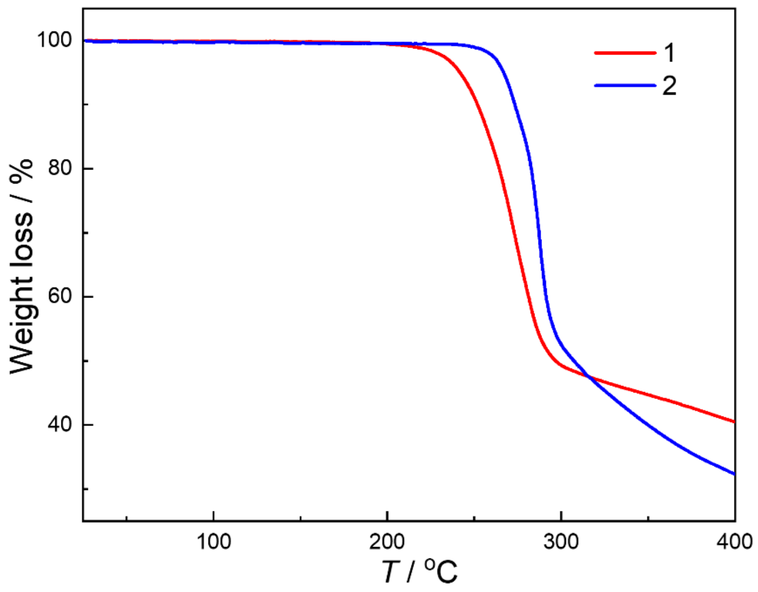

Figure S3. The TGA curves of **1** and **2** with a heating rate of 5 ℃/min^{-1} under N_2 flow.

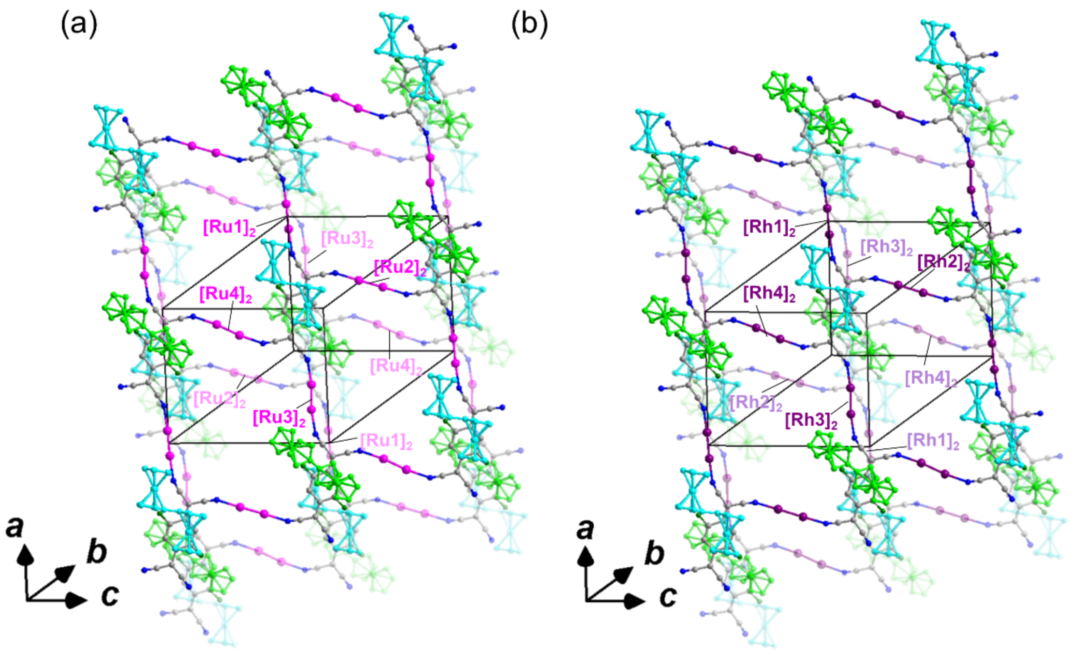

Figure S4. Packing views of **1** (a) and **2** (b) projected perpendicular to the (1−11) plane, in which hydrogen atoms and 2,3,5,6-$F_4ArCO_2^-$ ligands around the Ru/Rh centers are omitted for clarity, and C, N, Ru and Rh atoms are represented in gray, blue, purple, and violet, respectively. Subunits bifc-1 and bifc-2 are colored in cyan and yellow-green, respectively.

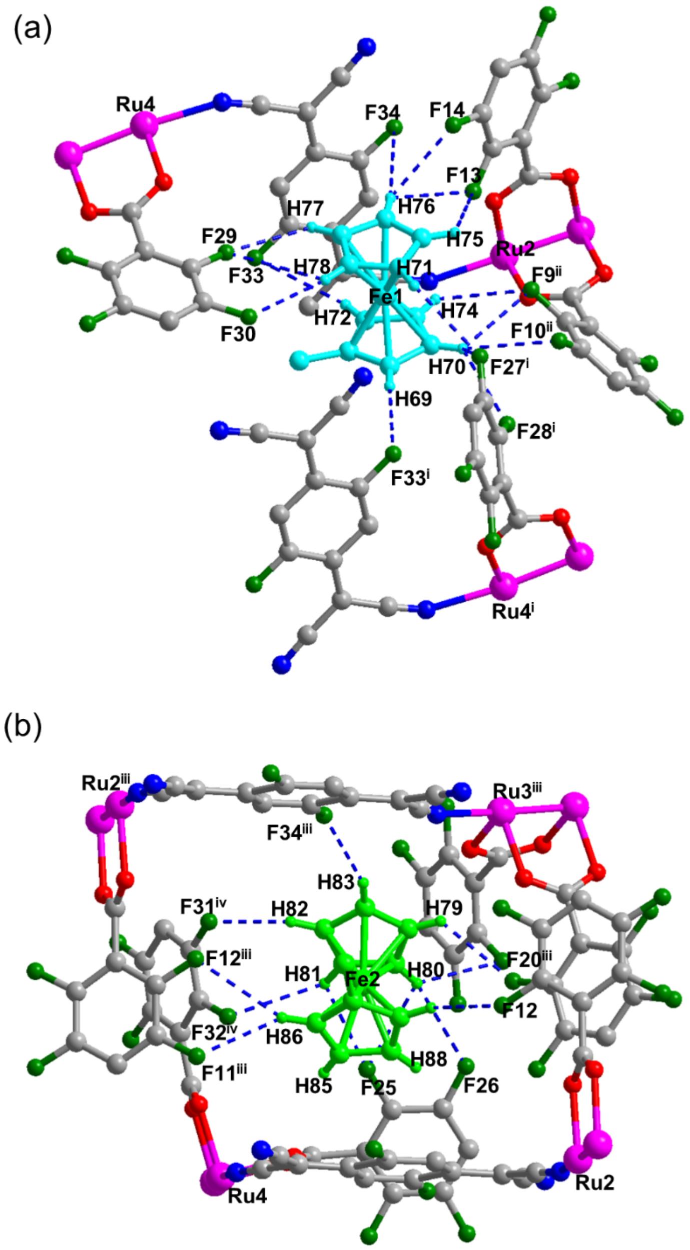

Figure S5. Packing diagrams of **1** representing C−H···F type hydrogen bond around bifc-1 (a) and bifc-2 (b). The symmetry operations are (i) −*x*, −*y*, −*z* + 2, (ii) −*x* + 1, −*y*, −*z* + 1, (iii) −*x* + 2, −*y* + 1, −*z* + 1, (iv) −*x* + 1, −*y* + 1, −*z* + 2.

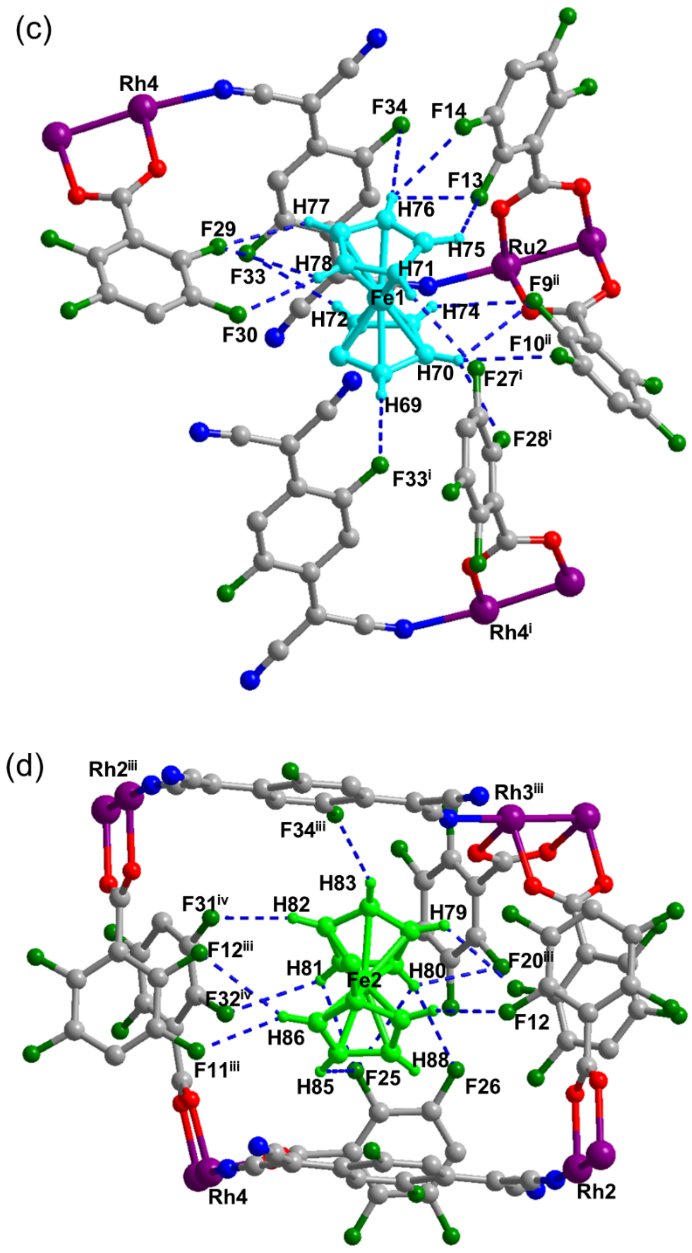

Figure S5 (continue). Packing diagrams of **2** representing C−H···F type hydrogen bond around bifc-1 (c) and bifc-2 (d). The symmetry operations are (i) $-x$, $-y$, $-z + 2$, (ii) $-x + 1$, $-y$, $-z + 1$, (iii) $-x + 2$, $-y + 1$, $-z + 1$, (iv) $-x + 1$, $-y + 1$, $-z + 2$.

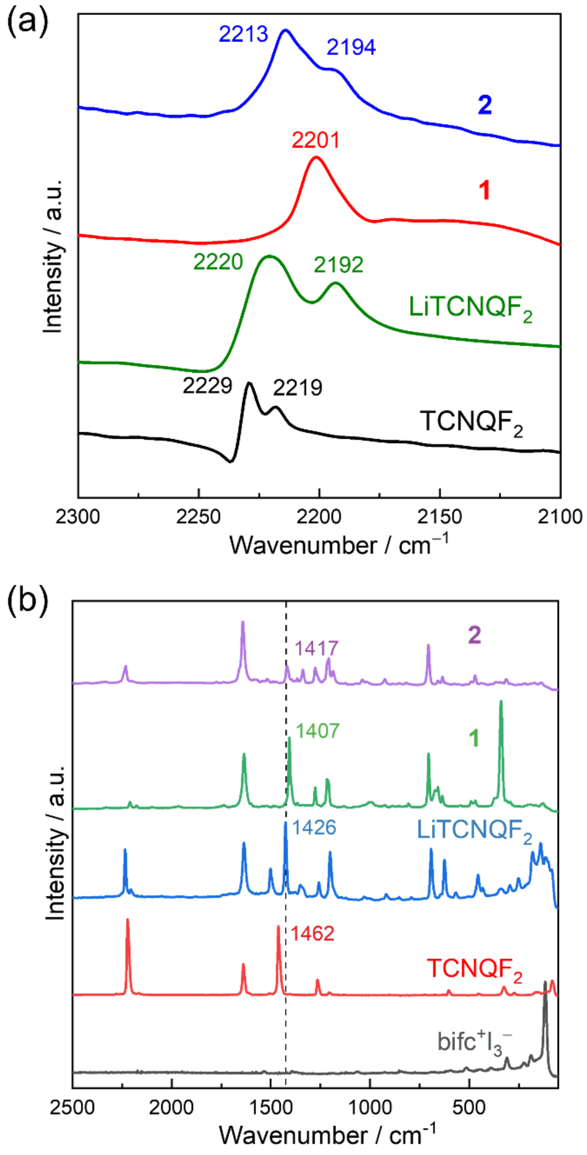

Figure S6. Infrared (a) and Raman (b) spectra of **1**, **2**, $TCNQF_2$, $LiTCNQF_2$, and $bifc^{+}I_3^{-}$ measured at room temperature.,

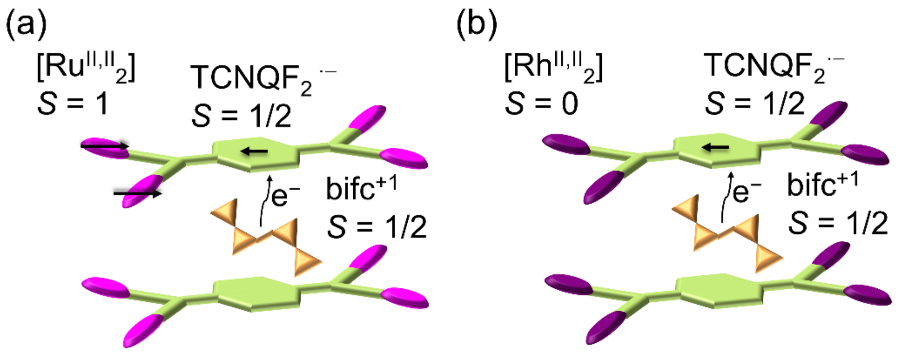

Figure S7. Electronic and spin states of assembly units and charge transfer in compounds **1** (left) and **2** (right). $[Ru_2^{II,II}(2,3,5,6\text{-}F_4ArCO_2)_4]$, pink; $[Rh_2^{II,II}(2,3,5,6\text{-}F_4ArCO_2)_4]$, purple; $TCNQF_2^{\bullet-}$, green; bifc$^+$, orange.

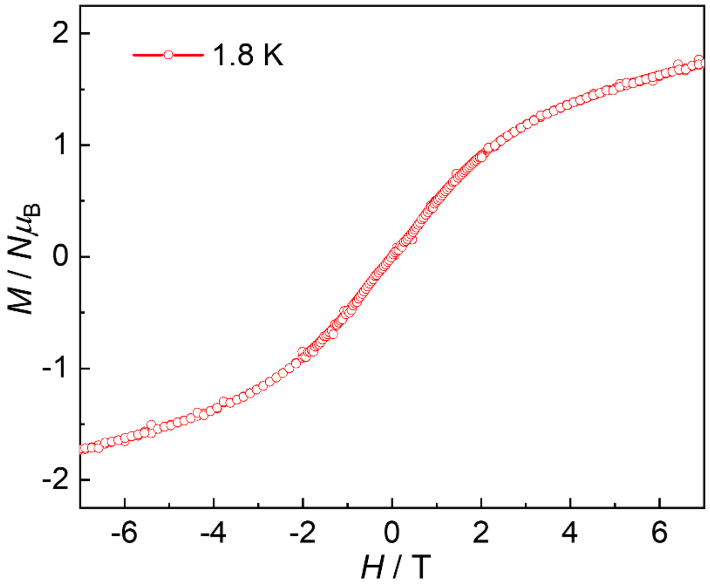

Figure S8. Magnetic field dependence of magnetization at 1.8 K of **2**.

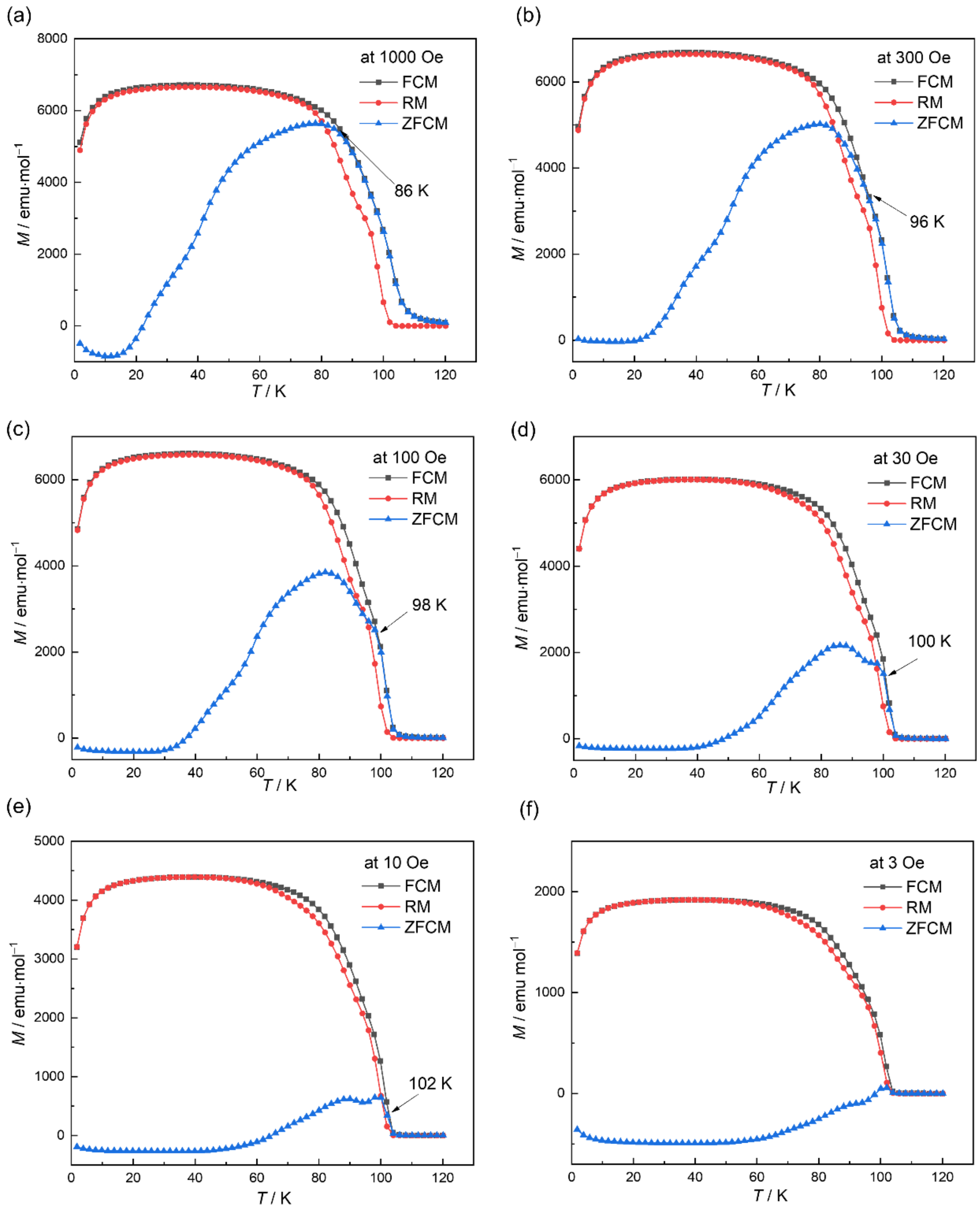

Figure S9. Field-cooled magnetization (FCM), zero-field-cooled magnetization (ZFCM), and remnant magnetization (RM) curves **1** measured from 120 K to 1.8 K under different dc fields. The diverged point of ZFCM and FCM curves (black arrow) showed the obvious shift.

Relaxation time analysis of spin glass behavior using Arrhénius law and the critical scaling model.

From the Figure 4a, we can see that χ" is significantly frequency dependent. Frequency-dependented slow relaxation behavior of the magnetization is usually observed in spin-glass, superparamagnetic, or long-range ordering systems. In order to confirm the relaxation behaviors in **1**, dynamic slowing analysis is necessary.

The critical scaling approach is a good model to analyze frequency dependence of freezing temperature in spin-glassy systems, $\tau = \tau_0 \cdot [\frac{T_B}{T_{SG}} - 1]^{-z\nu}$ (T_{SG} is the spin glass temperature when *f* is equal to zero, $z\nu$ is the dynamic critical exponent and T_B was defined as the value at the maximum point of relaxation peaks in $\chi''-T$ plot after multiple peak fit with Gauss function in Origin Pro2018.[20] The peak separation results are shown in Figure S9 and Table S7. Here, positions of fit peak 1 were almost unchanged, while fit peak 2 and 3 shifted to higher temperature with the increasing frequency. The fitting result gives reasonable parameters (Figure 4b), that is, τ_0, $z\nu$ and T_{SG} are 2.6×10^{-14} s, 7.1, and 91.9 K for 1st and 2.7×10^{-9} s, 6.9, and 73.6 K for 2nd relaxation, respectively. From this analysis, the value of $z\nu$ is in the typical range of a spin glass system between 4 and 12.[21] The τ_0 order of magnitude of 1st is larger than canonical spin glass (10^{-12}–10^{-13}),[22,23] but it is close to the spin cluster-glass behavior.[11] Generally, the reasonable fitting results suggest that both relaxation behaviors below 95 K in ac susceptibility are ascribed to spin-glassy characterized relaxation.

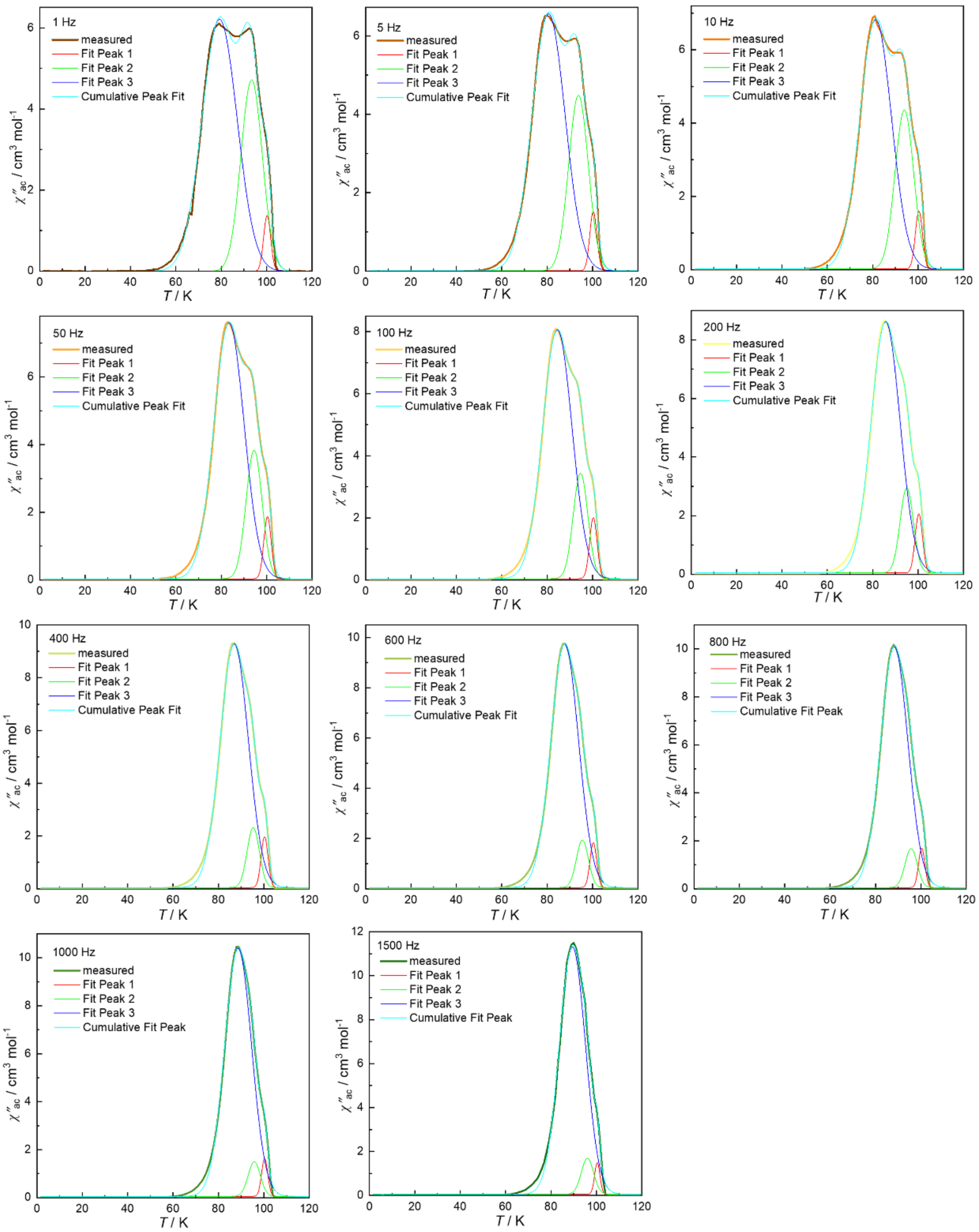

Figure S10. Multiple peak fit results of χ" at different frequency with Gauss function using Origin Pro 2018.

Table S7. χ'' peak positions of fit peaks after separation with Gauss function.

Frequency / Hz	Positon of fit peak 1 / K	Positon of fit peak 2 / K	Positon of fit peak 3 / K
1	100.2097(0.12466)	93.38435(0.15362)	79.22829(0.21492)
5	100.33862(0.09778)	93.79749(0.12041)	80.60765(0.16164)
10	100.36099(0.10042)	93.95487(0.12311)	81.30005(0.15651)
50	100.37244(0.11869)	94.46278(0.14218)	83.41586(0.15357)
100	100.34956(0.13849)	94.71234(0.16486)	84.48457(0.15148)
200	100.33933(0.16261)	95.01077(0.20347)	85.69213(0.15425)
400	100.33255(0.18557)	95.33041(0.26834)	86.956(0.16159)
600	100.34391(0.20103)	95.53876(0.34148)	87.7146(0.17328)
800	100.36891(0.20768)	95.71389(0.42198)	88.25271(0.19042)
1000	100.35502(0.21674)	95.78558(0.51374)	88.65838(0.2078)
1500	100.38836(0)	96.05628(0.44246)	89.42474(0.22595)

Stepwise relaxation behavior in 1.

For the one-electron-transferred layered D_2A-MOFs, their magnetic ground are related to the interunit distance between layers, translational distance (l_T). The AF/F boundary is determined at approximately l_T = 10.3 Å: l_T > 10.3 Å for ferromagnetic phase and l_T < 10.3 Å for antiferromagnetic phase.[24,25] Based on this rule, the closer interlayer distance will induce AF. The hypothesis of easy axis perpendicular to D_2A layer conflict the molecule dipole-dipole interaction, that is, same charge will repel. Therefore, their easy axis of magnetic anisotropy should be along the layer.

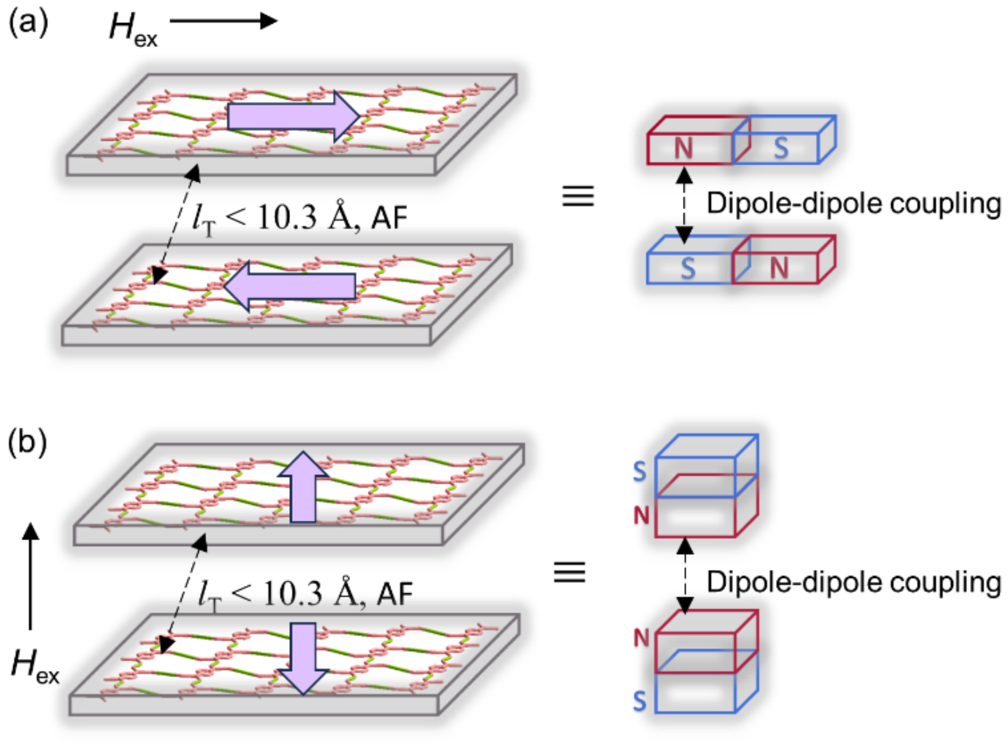

Figure S11. Schematic representations of spin alignments in the two-dimensional one-electron-transferred D_2A-typed antiferromagnetic phase: spin orientation is parallel (a) or perpendicular (b) to the fishnet layer.

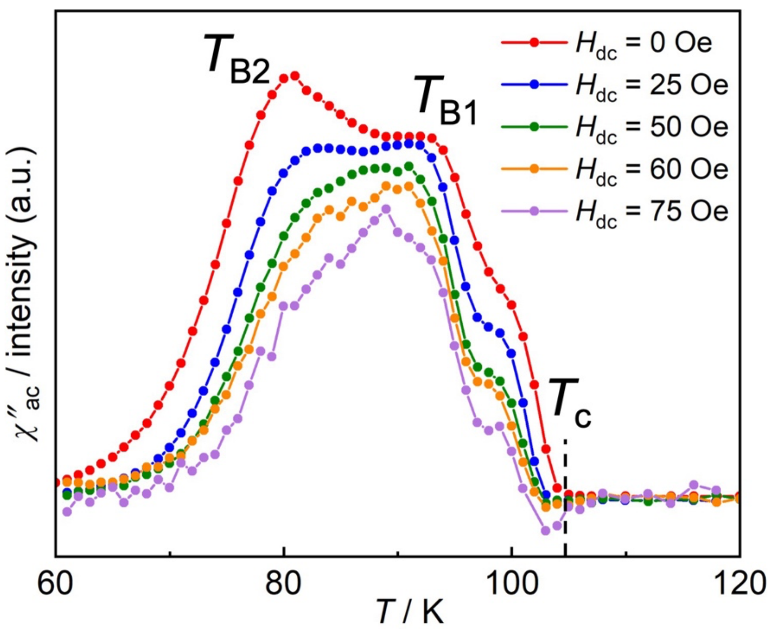

Figure S12. Temperature dependence of the AC magnetic susceptibility (out of phase) of **1** measured under several external DC fields with a 3 Oe oscillating field at a fixed frequency of 10 Hz.

References in SI

1 Y. Inagaki and M. Naya, European Patent EP1130585, 2001.
2 H. Miyasaka, N. Motokawa, R. Atsuumi, H. Kamo, Y. Asai and M. Yamashita, *Dalton Trans.*, 2011, **40**, 673-682.
3 G. A. Rempel, P. Legzdins, H. Smith and G. Wilikson, in *Inorganic Syntheses*, ed. F. A. Cotton, McGraw-Hill, Inc., New York, 1972, vol. 13, pp. 90-91.
4 W. H. Morrison and D. N. Hendrickson, *Inorg. Chem.* 1975, **14**, 2331-2346.
5 M. D. Rausch, *The Journal of Organic Chemistry*, 1961, **26**, 1802-1805.
6 K.-J. Chen, A. C. Tan, C.-H. Wang, T.-S. Kuo, P.-L. Chen and M. Horie, *Cryst. Growth Des.*, 2019, **19**, 17-22.
7 G. A. Bain,; J. F. Berry, , Diamagnetic Corrections and Pascal's Constants. *J. Chem. Educ.* 2008, **85**, 532.
8 O. V. Dolomanov, L. J. Bourhis, R. J. Gildea, J. A. Howard and H. Puschmann, *J. Appl. Crystallogr.*, 2009, **42**, 339-341.
9 G. Sheldrick, *Acta Crystallographica Section A*, 2008, **64**, 112-122.
10 G. Sheldrick, *Acta Cryst.*, 2015, **71**, 3-8.
11 H. Putz and K. Brandenburg GbR, DIAMOND, Bonn, Germany, 2019.
12 S. Furukawa and S. Kitagawa, *Inorg. Chem.*, 2004, **43**, 6464-6472.
13 F. A. Cotton and R. A. Walton, *Multiple Bonds Between Metal Atoms*, 2nd ed.; Oxford University Press: Oxford, 1993.
14 T. J. Kistenmacher, T. J. Emge, A. Bloch and D. Cowan, *Acta Crystallographica Section B: Structural Crystallography and Crystal Chemistry*, 1982, **38**, 1193-1199.
15 R. E. Long, R. A. Sparks and K. N. Trueblood, *Acta Crystallographica*, 1965, **18**, 932-939.
16 A. Hoekstra, T. Spoelder and A. Vos, *Acta Crystallographica Section B: Structural Crystallography and Crystal Chemistry*, 1972, **28**, 14-25.
17 W. Kosaka, Y. Takahashi, M. Nishio, K. Narushima, H. Fukunaga and H. Miyasaka, *Adv. Sci.*, 2018, **5**, 1700526.
18 H. Fukunaga and H. Miyasaka, *Angew. Chem.*, 2015, **127**, 579-583.
19 H. Fukunaga, W. Kosaka, H. Nemoto, K. Taniguchi, S. Kawaguchi, K. Sugimoto and H. Miyasaka, *Chem. Eur. J.*, 2020, **26**, 16755-16766.
20 Origin(Pro), Version *2018*. OriginLab Corporation, Northampton, MA, USA.
21 V. K. Anand, D. T. Adroja and A. D. Hillier, *Phys. Rev. B*, 2012, **85**, 014418.
22 J. Kroder, J. Gooth, W. Schnelle, G. H. Fecher and C. Felser, *AIP Advances*, 2019, **9**, 055327.
23 S. Pakhira, C. Mazumdar, R. Ranganathan, S. Giri and M. Avdeev, *Phys. Rev. B*, 2016, **94**, 104414.
24 H. Miyasaka, *Bull. Chem. Soc. Jpn.*, 2021, **94**, 2929-2955.
25 W. Kosaka, Z. Liu and H. Miyasaka, *Dalton Trans.*, 2018, **47**, 11760-11768.
